# Stretchable and colorless freestanding microwire arrays for transparent solar cells with flexibility

**DOI:** 10.1038/s41377-019-0234-y

**Published:** 2019-12-12

**Authors:** Sung Bum Kang, Ji-Hwan Kim, Myeong Hoon Jeong, Amit Sanger, Chan Ul Kim, Chil-Min Kim, Kyoung Jin Choi

**Affiliations:** 1 Department of Materials Science and Engineering, Ulsan National Institute of Science and Technology (UNIST), Ulsan, 44919 Republic of Korea; 2 Department of Emerging Materials Science, Daegu Gyeongbuk Institute of Science and Technology (DGIST), Daegu, 42988 Republic of Korea

**Keywords:** Solar energy and photovoltaic technology, Electronics, photonics and device physics

## Abstract

Transparent solar cells (TSCs) are emerging devices that combine the advantages of visible transparency and light-to-electricity conversion. Currently, existing TSCs are based predominantly on organics, dyes, and perovskites; however, the rigidity and color-tinted transparent nature of those devices strongly limit the utility of the resulting TSCs for real-world applications. Here, we demonstrate a flexible, color-neutral, and high-efficiency TSC based on a freestanding form of *n*-silicon microwires (SiMWs). Flat-tip SiMWs with controllable spacing are fabricated via deep-reactive ion etching and embedded in a freestanding transparent polymer matrix. The light transmittance can be tuned from ~10 to 55% by adjusting the spacing between the microwires. For TSCs, a heterojunction is formed with a p-type polymer in the top portion of the n-type flat-tip SiMWs. Ohmic contact with an indium-doped ZnO film occurs at the bottom, and the side surface has an Al_2_O_3_ passivation layer. Furthermore, slanted-tip SiMWs are developed by a novel solvent-assisted wet etching method to manipulate light absorption. Finite-difference time-domain simulation revealed that the reflected light from slanted-tip SiMWs helps light-matter interactions in adjacent microwires. The TSC based on the slanted-tip SiMWs demonstrates 8% efficiency at a visible transparency of 10% with flexibility. This efficiency is the highest among Si-based TSCs and comparable with that of state-of-the-art neutral-color TSCs based on organic–inorganic hybrid perovskite and organics. Moreover, unlike others, the stretchable and transparent platform in this study is promising for future TSCs.

## Transparent solar cells: Microwire-array shows potential

A flexible, stretchable and fully transparent solar cell shows promise for harvesting sunlight as it hits windows. In designing transparent solar cells, there is a trade-off between efficiency and transparency – many existing cells are rigid and tinted because of the way they absorb certain wavelengths of light, and this limits their use in electronic devices and windows. Now, Kyoung Jin Choi at the Ulsan National Institute of Science and Technology, Ulsan, Korea, and co-workers have used a freestanding form of microwire-arrays to create a flexible, fully transparent solar cell. Their design comprises a crystalline silicon microwire array embedded in a transparent polymer matrix. The conducting junction occurs where the n-type-Si microwire tips meet the p-type polymer. The team also manipulated the shape of the microwire tips so that this enhances efficiency whilst maintaining transparency.

## Introduction

Transparent solar cells (TSCs) are emerging as building blocks for building-integrated power generation^1–4^. In this attractive concept of photovoltaics, there is an unavoidable trade-off between the energy generation (i.e., the photovoltaic conversion efficiency (PCE)) and the light admission (visible transparency). Therefore, based on the criteria for transparent solar cells, there is an inevitable compromise of the efficiency to achieve transparency. The most common way to develop transparent solar cells is via band-gap engineering of active materials that can absorb sunlight selectively, resulting in tinted transparency^5–9^. For example, when the active layers are designed to absorb short-wavelength light and transmit long-wavelength light in the visible range (*λ* > 600 nm), the transparent devices exhibit a yellow or reddish color^7–14^. Previous attempts have been made to develop transparent solar cells by taking advantage of dyes as active materials. To obtain fully transparent dye-sensitized solar cells, a new design of dye sensitizers that ensures the absorption of visible light in the device may be effective^15,16^. Moreover, all components (including TiO_2_ and the electrodes) should also be transparent^17–19^. Through these strategies, some groups have demonstrated various tinted transparent solar cells with a low PCE of ~3−7% with modest transparencies^15–19^. However, this tinted transparency is unsuitable for electronics, automobile windows, and office-building-integrated photovoltaic windows.

Recently, there has been flurry of interest in the field of photovoltaics, focused on organic–inorganic hybrid perovskite materials^20–23^. These ABX_3_-structured materials exhibit a suitable band gap and high-absorption coefficient, making them an intriguing class of photovoltaics^24–28^. Many groups have achieved halide perovskite-based transparent solar cells by controlling the thickness, transport layer, and composition of the perovskite. Introducing a thin layer with a band-gap engineered perovskite layer is a well-known technique for achieving transparent solar cells^29,30^. Roldán-Carmona et al.^9^ utilized common methylammonium lead iodide as an absorber, with variations in the thickness, and obtained a PCE of 6.4%. Moreover, Jung et al. and Heo et al.^8,31^ employed modified hole or electron transport layers in their devices and demonstrated perovskite-based semi-transparent solar cells with a PCE of over 10%. However, despite their high PCE, such devices have tinted transparency, again due to the compromise on color. Generally, because a perovskite is designed to absorb a part of the visible range of the solar spectrum as the active layer of transparent solar cells, the devices exhibit brown-yellow transparency. In addition, approaches for obtaining transparency are restricted because thinning the active layer or engineering the band gap is highly dependent on the materials^5,7,12,14^. Thus, the adjustable range of the transparency of the resulting devices is very limited. On the other hand, high-efficiency per transparency has also been achieved with organic-based transparent solar cells by taking advantage of designed semiconducting polymers. However, like the case with dyes and perovskites, it is difficult to obtain spectrally flat absorption across the entire visible spectrum with tailored polymers, which is a requirement for neutral-color transparent solar cells. Therefore, although Cui et al.^32^ obtained a PCE of 8.38% with a visible transparency of 25.7% using an ultralow-band-gap nonfullerene acceptor, the device was only transparent in the blue-green region of the visible spectrum. Liu et al.^33^ developed a new electron-acceptor material that shows strong Near-infrared (NIR) absorption between 600 and 940 nm, and successfully applied it to transparent solar cells with blue-tinted transparency. Again, the tinted transparency of perovskite and organic-based TSCs is a major obstacle to real-world applications.

Herein, we demonstrate true-color transparent solar cells. A crystalline n-Si microwire array with controllable spacing is fabricated via deep-reactive ion etching and embedded within a transparent polymer matrix. Subsequently, via a combination of dry and wet etching, a freestanding Si microwire array polymer composite film (SiMPF) is obtained by applying mechanical peel-off techniques. Furthermore, we apply a p-type conductive polymer on top of the n-Si tips, enabling the formation of a junction between the polymer and n-Si, and fabricate a neutral-color transparent solar cell. The slanted-tip of the n-SiMPF-based transparent solar cells leads to a power conversion efficiency of 8.07% at a visible transparency of 10% with flexibility. The developed devices have performances comparable with those of existing TSCs based predominantly on perovskites, dyes, and organics. Moreover, this robust, ultra-light and stretchable platform is promising for future transparent and stretchable solar cells to extend their applications.

## Results

### Fabrication of freestanding Si microwire–poly(dimethylsiloxane) (PDMS) composite

**Fig. 1:**
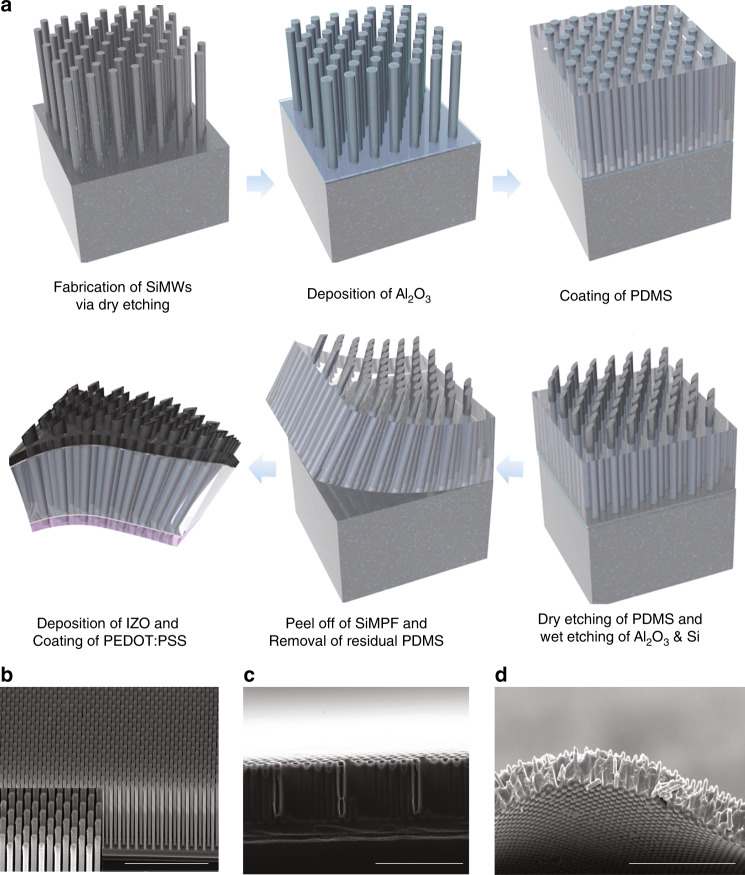
Experimental procedure of SiMPF composite. **a** Schematic of fabrication process. SEM images of **b** Si microwire arrays in a bird’s-eye view (scale bar: 50 μm), **c** Si microwire array filled with PDMS after surface treatment (scale bar: 50 μm), and **d** freestanding SiMPF after peeling off from the Si parent (scale bar: 100 μm).

Figure 1a displays the overall process for the fabrication of the TSCs based on the freestanding film of an n-type SiMW array embedded in poly(dimethylsiloxane) (PDMS). As shown in Fig. 1b, a hexagonal array of microwires with a diameter of 2 μm and a length of 30 μm was fabricated by a photolithography process involving reactive ion etching (RIE) using a Cr dot array as the etching mask. Second, a 15-nm-thick Al_2_O_3_ layer was deposited on the SiMWs by atomic layer deposition (ALD) to passivate the Si surface. Compared to conventional silicon solar cells, the freestanding SiMPF-based TSCs in this study have a very high surface area; thus, proper surface passivation is essential. As shown in Fig. S1 (Supplementary Information), the TSC without Al_2_O_3_ passivation had a very low short-circuit current density (*J*_sc_) and open-circuit voltage (*V*_oc_) due to the very high leakage current caused by surface defects. Third, the SiMW array was embedded in PDMS via spin-coating. PDMS embedding was performed by a two-step spin-coating process consisting of first spinning at 600 rpm for 120 s and then spinning at 1500 rpm for 10 s. In the first step, PDMS conformally and densely filled the SiMW array; the excess PDMS residue on the top of the SiMWs was removed during the second spin-coating step. We found that the Al_2_O_3_-coated SiMW array was so hydrophobic that PDMS could not deeply penetrate the SiMW, and the adhesion between SiMW and PDMS was not very strong. Thus, PDMS was easily peeled off the SiMW array in the second spin-coating step (Fig. S2a, b, Supplementary Information). The surface of the Al_2_O_3_-coated SiMWs was subjected to O_2_-plasma treatment, which enhanced the adhesion between the Al_2_O_3_ surface and PDMS due to covalent bonding of O–Si^34–36^. Figure 1c presents an scanning electron microscope (SEM) image of the SiMW array embedded in PDMS, and it can be seen that PDMS deeply penetrated the SiMW array after the oxygen plasma treatment. Fourth, the residual PDMS layer was removed by dry etching using a gaseous mixture of O_2_ and SF_6_. PDMS could be selectively etched because the SiMWs were protected by the Al_2_O_3_ film, which was very slowly etched by SF_6_ gas. Fifth, the SiMW array embedded in PDMS was peeled off the Si wafer, producing a flexible SiMPF as shown in Fig. 1d. The pitch and hexagonal arrangement of the SiMW array were maintained even after peeling off from the parent substrate, as manifested by the hexagonal array of diffraction spots when SiMPF was perpendicularly illuminated with a 532-nm diode laser (Fig. S3 and Movie S1, Supplementary Information). Finally, the TSC process was completed by forming a hetero p–n junction with PEDOT:PSS at the exposed tips and the indium-doped zinc oxide (IZO) ohmic contact at the bottom of the SiMPF.

After the pitch, defined as the center-to-center distance of the microwire, was varied from 4 to 7 μm at 1 μm intervals (Fig. S4, Supplementary Information). Figure S5a, b show the current density (J)–voltage (V) characteristics and external quantum efficiencies (EQEs) of the TSCs based on flat-tip SiMW arrays. A summary of the device characteristics is presented in Table 1. Moreover, the box plot of *J*_sc_, *V*_oc_, and the efficiency of eight solar cells fabricated at pitch has a fairly narrow distribution, indicating that this fabrication process is fairly reproducible, as shown in Fig. S6. Compared to bulk solar cells, nanowire or microwire solar cells typically have a very high surface area and, consequently, a high concentration of surface defects that act as leakage current paths^37,38^. However, the deposited Al_2_O_3_ layer provides a high density of negative charges with a very low-density of interface defects^39–41^. Thus, the surface defects and dangling bonds can be effectively passivated. In addition, the presence of negative charges at the interface with silicon avoids parasitic shunting^42^.

**Table 1:** Average photovoltaic performance from 8 samples of flat-tip SiMPF-based transparent solar cells with controlled spacing between microwires.

Pitch (μm)	*V*_oc_ (V)	*J*_sc_ (mA/cm^2^)	FF	Eff. (%)
4	535 (542)	17.75 (18.3)	67.5 (68.1)	6.41 (6.75)
5	519 (522)	11.34 (12.24)	65.0 (65.7)	3.83 (4.19)
6	485 (493)	9.18 (9.54)	59.8 (60.4)	2.66 (2.84)
7	452 (456)	7.12 (7.74)	55.8 (56.2)	1.8 (1.98)

Values in the brackets are the photovoltaic performance from the champion device

Therefore, as shown in Fig. S5a and Table 1, the photovoltaic parameters of the 4-μm pitch cell were 0.542 V and 68.1%, which are comparable to those of the bulk counterparts. This means that the heterojunction between the n-type Si and p-type PEDOT:PSS works as an excellent rectifying junction, and the Al_2_O_3_ layer also efficiently passivates the silicon surface defects. As the pitch of the SiMW increased, the *J*_sc_ of the TSCs decreased, which can be explained by the decrease in the optical absorption. As the pitch increased from 4 to 7 μm, the average optical absorption of the TSCs at wavelengths of 400−1100 nm was reduced from 78.11 to 38.71% (Table 2), which is in good agreement with the reduction in the *J*_sc_. Similar to the change in the *J*_sc_, the *V*_oc_ is also inversely proportional to the optical transmittance because the *V*_oc_ of a conventional solar cell is related to the photocurrent (*I*_L_) and the saturation current (*I*_0_) as follows: *V*_oc _~ ln (*I*_L_/*I*_0_ + 1)^43,44^. Owing to the dependency of *V*_oc_ and *J*_sc_ on the optical transparency, the efficiency of the TSCs also has a trade-off relationship with the optical transparency, which is inevitable in TSCs.

**Table 2:** Optical properties (reflectance, transmittance) of flat-tip SiMPF.

Flat averaged reflectance				
Pitch (μm)	4	5	6	7
Visible + NIR (400–1100 nm)	11.84	13.90	12.02	10.48
Visible (400–800 nm)	10.10	11.60	10.57	9.44
Near-IR (800–1100 nm)	14.22	17.05	14.01	11.90
Averaged transmittance				
Pitch (μm)	4	5	6	7
Visible + NIR (400–1100 nm)	10.05	26.85	39.95	50.81
Visible (400–800 nm)	10.23	24.95	38.53	50.28
Near-IR (800–1100 nm)	9.80	29.46	41.89	51.52
Averaged absorbance				
Pitch (μm)	4	5	6	7
Visible + NIR (400–1100 nm)	78.11	59.24	48.03	38.71
Visible (400–800 nm)	79.66	63.45	50.90	40.28
Near-IR (800–1100 nm)	75.98	53.49	44.10	36.58

### Enhancing the light absorption of SiMPF via engineering light absorption

**Fig. 2:**
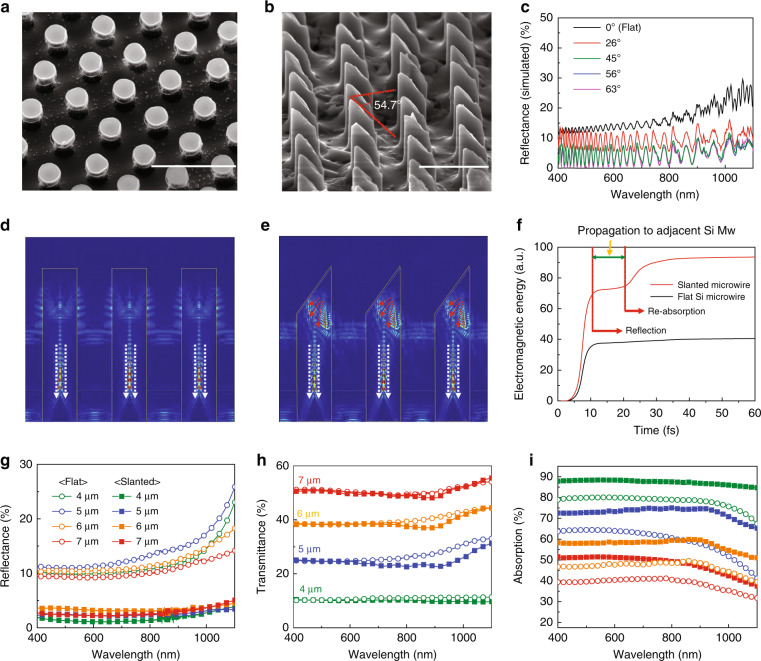
Enhancing the light absorption of SiMPF by etching. **a** SEM image of flat-tip SiMPF without etching (scale bar: 10 μm). **b** Slanted-tip SiMPF with etching by HNAD solution having a HF: HNO_3_: CH_3_COOH: DMF volume ratio of 3:6:0.3:0.7 (scale bar: 5 μm). **c** Simulated reflectance of SiMPF with difference angle of etched surface. The electric field intensity in the **d** flat and **e** slanted-tip SiMPF. **f** The total amount of electromagnetic energy in a single flat-tip SiMPF (black solid line) and slanted-tip SiMPF (red solid line). **g** Transmittance spectra of the slatned SiMPF of different center-to-center distance between microwires. **h** Reflectance spectra of the flat-tip (circle dot) and slanted-tip SiMPF (square dot) as a function of wavelength. **i** The total amount of absorption in slanted-tip SiMPF: 4 μm (green line), 5 μm (blue line), 6 μm (yellow line), and 7 μm (red line).

Nanowire arrays are known to have excellent light antireflection properties due to their graded refractive index effects and light-trapping ability^45–48^. Thus, nanowire arrays can have lower reflection than microwire arrays. However, nanowire arrays are manufactured by top-down methods^49–51^, including electron beam lithography and subsequent etching, or bottom-up methods^52–54^, including the vapor–liquid–solid method. Both methods have very limited scalability of the overall area. Moreover, these arrays have a very high surface-to-volume ratio, which increases the possibility of surface recombination and degrades performance^38,55,56^. In contrast, wafer-scale microwire arrays are manufactured by photolithography and etching processes that are now widely used in the semiconductor industry. In addition, microwire arrays are advantageous for forming effective junctions, allowing for easy carrier collection^37,57^. Therefore, SiMWs with reduced reflectance can be a promising candidate for TSCs. Assuming that the reflectance of the SiMWs is simply proportional to the area of the top flat surface of the microwire array, the average calculated reflectance obtained with the SiMWs having a pitch of 4 μm was as high as 9.54% (Fig. S7a, Supplementary Information). Thus, to reduce the high reflectance from the flat-tip SiMWs, anti-reflective (AR) coatings or surface texturing techniques, which are typically adopted in bulk silicon solar cells, can be applied. First, we fabricated a TSC with a 70-nm-thick SiN AR coating on the SiMW tips deposited by plasma-enhanced chemical vapor deposition (PECVD). Figure S7b shows the *J*–*V* characteristics of the TSC based on the 4-μm-pitched SiMW arrays with SiNx. With the use of the antireflection film, the *J*_sc_ increased from 17.07 to 18.94 mA/cm^2^, but the *V*_oc_ and FF decreased simultaneously, resulting in no significant improvement in the efficiency compared to that of the TSC without the SiN layer. The reduction in the *V*_oc_ can be attributed to the fact that the n-Si and p-PEDOT heterojunctions are formed only on the side surface, not on the top surface of the SiMWs, thereby failing to efficiently separate the electron-hole pairs. Thus, attempts were made to reduce the light reflection by changing the shape of the flat-tip SiMWs (Fig. 2a). For this purpose, a 70-nm-thick SiO_2_ thin film was formed on the tips of the SiMWs as an etching mask, and the SiMWs were chemically etched using a hydrofluoric acid−nitric acid–acetic acid (HNA) mixture solution^58–61^. Silicon etching using the HNA solution proceeds in three steps as follows:

1 $${\mathrm{Si}} + 2{\mathrm{H}}^ + \to 2{\mathrm{Si}}^{2 + } + {\mathrm{H}}_2$$

2 $${\mathrm{Si}}^{2 + } + 2{\mathrm{OH}}^ - \to {\mathrm{Si}}\left( {{\mathrm{OH}}} \right)_2 \to {\mathrm{SiO}}_{2 + }{\mathrm{H}}_2$$

3 $${\mathrm{SiO}}_2 + 6{\mathrm{HF}} \to {\mathrm{H}}_2{\mathrm{SiF}}_6$$

The overall reaction is initiated by breaking the covalent bonds between the silicon atoms by H^+^ ions supplied from HNO_3_, producing SiO_2_ by recombination of Si^2+^ ions with hydroxide ions. Thereafter, HF dissolves the silicon dioxide to produce an aqueous salt. On the other hand, CH_3_COOH acts as a diluent to prevent excessive dissociation of HNO_3_ and to adjust the etching rate and the roughness of the etched surface. SiMPF was vigorously etched and even partially removed from the PDMS matrix in the HNA solution with a composition of a 3:7:0, whereas the etching rate was considerably attenuated in the HNA etching solution with a 3:6:1 composition ratio (Fig. S8a, b, Supplementary Information). However, the SiMWs were still extensively etched by the solution, leaving only the Al_2_O_3_ layer behind. To reduce the etching rate and change the behavior of HF-based etching, various additives, including organics, were introduced into the HF solution^61,62^. Surprisingly, the etching rate was significantly reduced with the etching solution in which Dimethylformamide (DMF) was added instead of acetic acid, and this dramatically changed the shape of the SiMW tip (Fig. S8c). The interaction between the HF and DMF molecules leads to the formation of heteroassociates. These heteroassociates have a pyramidal molecular structure consisting of three H–F bonds (H on DMF and F on HF), which leads to redistribution of the electron density of HF^63^, thereby greatly reducing the etching rate of SiO_2_. As a result, the etching rate of the native oxide (as an etching intermediate), as well as that of the SiO_2_ etching mask layer on the top surface of the SiMWs, is significantly reduced, which modifies the shape of the SiMW tips while maintaining the overall morphology of the SiMWs. Furthermore, we conducted a series of etching experiments while varying the volume ratio of the HF−HNO_3_−CH_3_COOH−DMF (HNAD) etching solution and found that the HNAD solution with a volume ratio of 30:60:3:7 generated SiMWs with well-defined, uniform, slanted tips, as shown in Fig. 2b. The measured angle of the slanted SiMW tips was ~54.7°. Simulation of the angle-dependent reflectance of the slanted-tip also shows that the reflectance decreased up to an angle of 45°, thereafter reaching saturation (Fig. 2c). Finite-difference time-domain (FDTD) simulations were conducted to study the reflection and absorption of light from the flat and slanted SiMW tips. Approximately 40% of visible and infrared light was reflected by SiMPF with the flat-tip; thus, the strength of the electric field inside the SiMWs was low (Fig. 2d). On the other hand, light incident to the slanted-tip of the SiMWs was reflected towards the inside of SiMPF and eventually re-absorbed by adjacent SiMWs, thereby increasing the intensity of the electric field inside the SiMWs. Moreover, the low reflectance of the slanted-tip SiMPFs is mainly due to re-absorption of reflected light from the slanted-tip by adjacent microwires (Fig. 2e). The time difference between the two absorption events provides evidence that reflected light from the slanted-tip MWs is re-absorbed by adjacent SiMWs. After 10.5 fs, the electromagnetic energy inside the SiMWs increases again, suggesting that the reflected light propagates to adjacent SiMWs for 10.5 fs. When multiplying by the group velocity of their pulse^64^, it corresponds to 3.17 µm. This is in good agreement with the distance between the center of the SiMWs to the edge side of the adjacent Si MW, indicating that reflected light from the slanted-tip is re-absorbed by adjacent wires (Fig. 2f). Therefore, the absorbed electromagnetic energy of the slanted-tip microwires consists of electric fields from the non-reflected light (white arrow, Fig. 2d) and the scattered electric field from re-absorbed light due to refracted light (red arrow, Fig. 2e). Figure 2g−i show the reflection, transmission, and absorption spectra of SiMPF with flat and slanted tips as a function of the pitch obtained by UV–Vis spectroscopy measurements. As predicted, the average reflectance decreased dramatically from 10.48 to 13.90% for the flat-tip SiMPFs to 1.81 to 3.45% for the slanted-tip counterparts (Table 3). Notably, the flat-tip SiMPF with a pitch of 4 μm or the highest density of microwires had the lowest reflectance. This can be explained by light-trapping effects from scattering and diffraction due to the narrow spacing (~2 μm) between the SiMWs^65,66^.

**Table 3:** Optical properties (reflectance, transmittance) of slanted-tip SiMPF.

Slanted averaged reflectance				
Pitch (μm)	4	5	6	7
Visible + NIR (400–1100 nm)	1.80	2.55	3.44	2.85
Visible (400–800 nm)	1.30	2.33	3.31	2.40
Near-IR (800–1100 nm)	2.49	2.85	3.62	3.46
Averaged transmittance				
Pitch (μm)	4	5	6	7
Visible + NIR (400–1100 nm)	10.87	24.65	39.03	50.33
Visible (400–800 nm)	10.61	24.08	38.28	49.89
Near-IR (800–1100 nm)	11.22	25.44	40.06	50.94
Averaged absorbance				
Pitch (μm)	4	5	6	7
Visible + NIR (400–1100 nm)	87.33	72.80	57.52	46.82
Visible (400–800 nm)	88.09	73.59	58.41	47.71
Near-IR (800–1100 nm)	86.29	71.72	56.32	45.60

The transmittance spectra of the flat and slanted-tip SiMPFs are predominantly determined by the pitch and were similar, except that the transmittance of the slanted samples was slightly lower than that of the planar sample in the long-wavelength regime. The light absorption coefficient of silicon is small due to the indirect band-gap structure and tends to decrease exponentially with increasing wavelength^67,68^. Therefore, the 30-μm-thick silicon layer used in this study may not completely absorb 1-sun illumination^69^. Time-resolved light-tracing simulation was performed at two wavelengths (short: 550 nm) and (long: 900 nm) to elucidate the origin of this difference in the transmittance of the flat and slanted samples. With short-wavelength incident light, the light intensity was rapidly attenuated as the depth of the microwire increased for both the flat and slanted samples, indicating that the short-wavelength light is fully absorbed by 30-μm-thick Si (Fig. S9a, b). On the other hand, with long-wavelength incident light on the flat-tip SiMWs, the light absorption was so weak that a significant portion of the light was transmitted through the bottom of the SiMW (Fig. S10a). However, when long-wavelength light was incident on the slanted-tip at an incidence angle of 50°, the light underwent refraction at the air-silicon interface, and thus the light inside the microwire followed a zigzag path (Fig. S10b), thereby increasing the effective light path or decreasing the transmittance (Fig. 2h). As a result, the absorption, calculated from the reflection and transmittance data, was significantly improved in the slanted-tip SiMPF (Fig. 2i). For example, the absorption in the SiMWs with a pitch of 5 μm increased (by 13.56%) from 59.24% for the flat-tip sample to 72.80% for the slanted-tip counterpart. The average reflectance, transmittance, and absorption of the flat and slanted-tip SiMPFs are summarized in Tables 2 and 3.

For the application of TSCs, the haze value is important because it can reduce clarity when viewing something through the TSC. The haze value represents light scattering and was calculated using the following equation:

4 $${\mathrm{Haze}}(\% ) = \frac{{T_{{\mathrm{diffuse}}}}}{{T_{{\mathrm{total}}}}} \times 100\%$$

where *T*_total_ is the total transmittance and *T*_diffuse_ is the diffuse transmittance (detailed measurement methods in Fig. S11 and Supplementary Information)^70,71^. As expected, the narrower the pitch between the microwires is, the higher the substantial degree of scattering. Thus, the sample with a lower pitch showed a higher haze ratio. For a narrow pitch of 4 µm, the haze values are relatively high, i.e., >15%. On the other hand, samples with a 7-µm pitch show very low haze values of ~2.5%, which are even comparable with those of indium tin oxide or ultra-thin silver nanowires, which have 1–3% haze values (Fig. S12a)^72–74^. Interestingly, the lower the pitch is, the larger the haze ratio difference between flat and slanted sample. The slanted-tip of the microwire allows the light inside the microwire to follow a zigzag path (Path "A" in Fig. S12b) or reflect towards an adjacent microwire (Path "B" in Fig. S12b). Light following path "A" is almost absorbed. On the other hand, the light in path "B" may cause secondary scattering to adjacent Si, resulting in the increased haze for the samples with particularly low pitch.

**Fig. 3:**
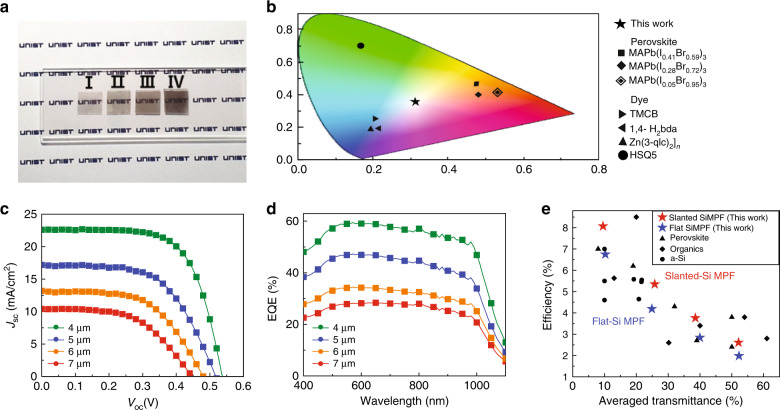
Performance of transparent solar cells based on the slanted-tip SiMPF. **a** Optical images of freestanding slanted-tip SiMPF. **b** The representation of the color coordinates of slanted-tip SiMPF (black star, this work), halide perovskites (black diamond, black square, and white diamond containing small black diamond), dyes (black right pointing triangle, black left pointing triangle, black up pointing triangle, and black circle) with AM1.5 G illumination on the CIE chromaticity diagram. **c** *J*–*V* characteristics and **d** EQE of slanted-tip SiMPF-based transparent solar cells: 4 μm (green line), 5 μm (blue line), 6 μm (yellow line), and 7 μm (red line). **e** The comparisons with previous reported neutral-color transparent solar cells based on halide perovskite, dye, a- Si, and organics.

Figure 3a presents an optical photograph showing the neutral-color perception of the SiMPFs with a controlled pitch, unlike the perovskite filter. Samples with pitches of 7 µm (I), 6 µm (II), 5 µm (III), and 4 µm (IV) are displayed on a university logo background. The transparency changed according to the pitch, but the color was perceived without distortion. For precise evaluation of the color perception, the SiMPF was illuminated with simulated AM1.5 light, and the color coordinates of the transmitted light were represented on the CIE 1931 chromaticity diagram, as shown in Fig. 3b and Table 4. For comparison, the color coordinates of various dyes commonly used in dye-sensitized solar cells (DSSCs) and MAPbI_*x*_Br_3−*x*_ perovskite films are also displayed. The perovskite thin films were fabricated in this study, and the data for the dyes with the DSSCs were referenced from other publications^16,75,76^. The dyes are green or blue-tinted, the perovskite films are reddish brown to yellowish (Fig. S13, Supplementary Information), and the SiMPF in this study has chromaticity coordinates in the central region of the chromaticity diagram.

**Table 4:** The color coordinates of dye, perovskite, and SiMPF represented in the CIE 1931 chromaticity diagram.

Materials	Color	CIE 1931		Ref.
		*x*	*y*	
Dye				
HSQ5	Green	0.68	0.19	[16]
TMCB	Blue	0.21	0.20	[76]
1,4-H_2_bda	Blue	0.19	0.21	[75]
Zn (3-qlc)_2_]_n_	Blue	0.19	0.17	[75]
Perovskites				
MAPb (I_0.05_Br_0.95_)_3_	Brown	0.53	0.42	This study
MAPb (I_0.28_Br_0.72_)_3_	Brown	0.47	0.40	This study
MAPb (I_0.41_Br_0.59_)_3_	Yellow	0.46	0.47	This study
SiMPF	Gray (neutral)	0.34	0.34	This study

### Performance of neutral-color transparent solar cells with flexibility

Figure 3c displays the *I*–*V* characteristics of the TSCs employing the slanted-tip SiMPFs, and the detailed photovoltaic performance is summarized in Table 5. Since HNAD etching only affects the upper part of the Si microwires already exposed in the first step, Al_2_O_3_ passivation on the sides of the microwire array still exists regardless of the flat or slanted-tip. Therefore, compared to the TSCs based on flat-tip SiMPFs, the *V*_oc_ and FF of the slanted devices remained unchanged, but *J*_sc_ increased significantly. In addition, the fabrication process including HNAD etching is still reproducible. As shown in Fig. S14, the box plot of the photovoltaic parameters of slanted-tip SiMPFs also has a narrow distribution. Interestingly, as the pitch of the SiMPFs increased, the enhancement in *J*_sc_ (Δ*J*_sc_) increased, which is in good agreement with the changes in the light absorption as a function of the pitch of the flat and slanted samples. The TSC with a pitch of 4 μm exhibited the highest efficiency of 8.07%. Figure 3d shows the EQE spectra of slanted TSCs with different pitches from 4 to 7 μm in the wavelength range of 400−1100 nm. The integrated *J*_sc_ is consistent with that determined from the *J*–*V* curves. Comparison of the EQEs of the flat and slanted samples shows a significant increase in the current density of the slanted samples in the near-infrared region, rather than in the visible region (Fig. S15, Supplementary Information). In other words, the enhancement in the efficiency of the TSCs is achieved by improving the absorption in the near-infrared region (800–1100 nm) without compromising the visible transparency, which is one of key parameters for TSCs.

**Table 5:** Average photovoltaic performance from 8 samples of slanted-tip SiMPF-based transparent solar cells with controlled spacing between microwires.

Pitch (μm)	*V*_oc_ (V)	*J*_sc_ (mA/cm^2^)	FF	Eff. (%)
4	533 (537)	21.84 (22.54)	66.1 (66.7)	7.70 (8.07)
5	511 (516)	16.77 (17.08)	60.1 (60.8)	5.15 (5.35)
6	476 (479)	12.81 (13.47)	57.3 (58.2)	3.49 (3.75)
7	445 (450)	9.98 (10.43)	54.6 (55.7)	2.42 (2.61)

Values in the brackets are the photovoltaic performance from the champion device

**Fig. 4:**
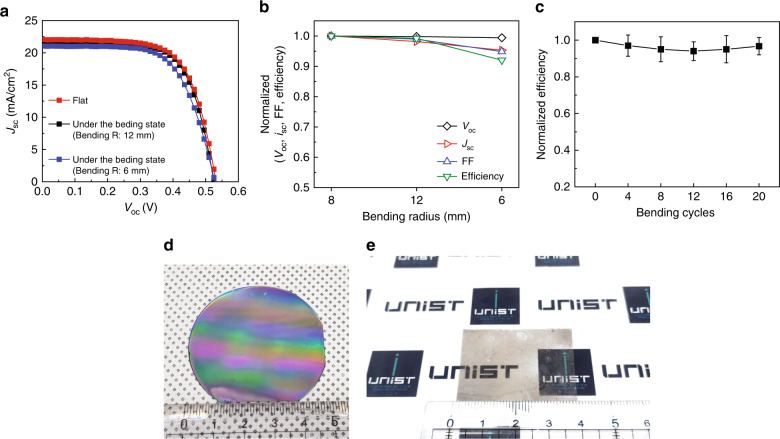
Flexibility of transparent solar cell and large scalability of freestanding SiMPF. **a** Light *J*–*V* curves and **b** normalized photovoltaic parameters of transparent solar cells under the bending state with the different bending radius. **c** Normalized efficiency of the transparent solar cells after cyclic bending test. Photographic images of **d** Si Mw arrays on 2-inch wafer, and **e** Freestanding SiMPF peeled from **d**.

Figure 3e shows a plot of the efficiency vs. the average transmittance of visible light for various neutral-color transparent solar cells, including those based on perovskites, organic semiconductors, and amorphous silicon. Zhang et al.^77^ demonstrated an ~9% neutral-color TSC by taking advantage of a NIR nonfullerene acceptor at a light transmittance of 20%. Chueh et al.^78^ fabricated a TSC with an efficiency of 5.63% at a light transmittance of 13% by reducing the thickness of the light-absorbing layer and the silver electrode in an organic solar cell. Song Yi et al.^79^ demonstrated a transparent organic solar cell in which the metal electrodes were replaced with graphene electrodes, achieving an efficiency of 3.8% at a visible transparency of 51%. Eperon et al.^80^ introduced microstructured perovskites and enabled the transmission of light through devices, achieving an efficiency of ~7% at a visible transparency of 8%. Alternatively, even when a thin layer of a-Si (<300 nm) is utilized for the active layer in TSCs^81^, the adjustable transparency range is very limited. When the transmittance is increased, an uncontrolled shunt path is formed in thin a-Si, resulting in serious efficiency loss, which further worsens the transmittance gain. Overall, the performance is comparable with that of the state-of-the-art TSCs based on organics or perovskite, but the transparency is low in comparison to TSCs that have high transmittance (*T* > 30%). To improve transparency, surface treatment of the PDMS that filled the spaces between the microwires can be further considered in future work. However, the transparency of the devices based on the SiMPF platform can be easily tuned from 10 to 55% by varying the spacing between the SiMWs, unlike other developed TSCs. More importantly, the TSC based on SiMPF can be applied as a flexible solar cell due to the thin thickness of the device and intrinsic robustness of PDMS^82–84^, whereas most of the currently reported TSCs utilizing organics or perovskite are fabricated by spin-coating on rigid glass, resulting in inflexibility. The TSC was characterized under the bending state and after a cyclic bending test. Interestingly, the performance of the transparent solar cells composed of PEDOT:PSS/Si MW–PDMS composite/IZO did not decreased severely in the bending state for bending radii of 12 mm and 6 mm (Fig. 4a, b). The photovoltaic parameters of the device in the bending state are summarized in Table 6. In addition, as shown in Fig. 4c, after the cyclic bending test with a bending radius of 6 mm, the normalized efficiency was almost maintained without a significant decrease, indicating that the transparent solar cell can be bent. Furthermore, SiMPF can be easily sized to wafer-scale unlike perovskite and organics, as shown in Fig. 4d, e, because it were fabricated using large-area processes, such as photolithography and dry etching.

**Table 6:** The photovoltaic parameters of transparent solar cells under the bending state with different bending radius.

Bending radius	*V*_oc_ (V)	*J*_sc_ (mA/cm^2^)	FF	Eff. (%)
∞ (Flat)	0.529	22.07	0.683	7.79
12 mm	0.528	21.88	0.676	7.81
6 mm	0.526	21.42	0.648	7.30

## Discussion

### Stretchability of the SiMPF platform and its application for stretchable solar cells

**Fig. 5:**
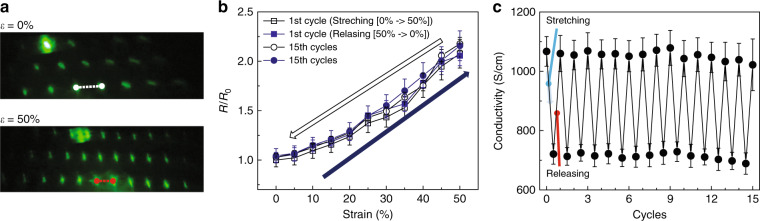
Stretchability of freestanding SiMPF. **a** Optical diffraction pattern of the SiMPF at *ε* = 0 and 50%. **b** The changes in resistances at each strain values as the 5% strain interval during stretching (0 → 50 %) and releasing state (50 → 0 %) at 1 cycle and 15 cycles. **c** Changes in conductivity when the applied strain is 0 and 50% under repetition of stretching and releasing cycles.

As shown in Fig. 5a, the optical diffraction patterns are produced by transmitted light, demonstrating the Fourier transform properties between the reciprocal domains. Under the application of 50% strain, the long-range order representing the reciprocal domains of the wire arrays was still observed, indicating that the morphology is maintained without any structural changes. After coating PEDOT:PSS on SiMPF before the deposition of IZO, we measured the changes in the resistances of PEDOT:PSS for each strain value at 5% strain intervals during stretching (0 → 50%) and releasing (50 → 0%) (Fig. 5b). After one cycle of stretching and releasing, the resistance of PEDOT:PSS coated on SiMPF recovered to the original value. Moreover, the changes in resistance after the 15th cycle are very similar to the value of the 1st cycle, indicating the durability of the samples. Moreover, under the application of a strain of 50%, the conductivity of the sample is maintained at up to ~700 S/cm, which is still a feasible value and applicable for photovoltaics. Additionally, this stretchable characteristic of robust SiMPF is retained during multiple cycles of strain and release (Fig. 5c). The stretchability of this platform can extend its applications to a solar window in a unique way. For example, under the application of strain, the pitch can be elongated at the same time. Therefore, as shown in Fig. S16, the transparency can be tuned as a function of strain, and it will also be applicable for a solar window that can control the transparency, allowing it to be used for both privacy and electricity generation depending on the situation.

Applying uniaxial or biaxial strain is a more severe condition for electronics than bending strain^85,86^. In the case of IZO, which is currently utilized as the bottom contact of transparent solar cells, it is not stretchable, but it is bendable. For stretchable solar cells, an EGain (eutectic gallium-indium)–Ag particle electrode was utilized, which exhibit no significant changes in sheet resistance under the application of strain, unlike IZO (Fig. S17). Under the application of strain, the stretchable solar cells still operate, but the performance deteriorates. The series resistance of the devices is increased due to the reduction in the conductivity of PEDOT:PSS under application of strain, resulting in a decrease in FF and *J*_sc_. However, after one cycle of stretching and release, the photovoltaic parameters are recovered due to the stretchable property of PEDOT:PSS/n-SiMPF (Fig. S18a). The integrated *J*_sc_ from the EQE spectra of the device under application of different strains in the wavelength range from 400 to 1100 nm is consistent with the *J*_sc_ determined from the light *J*–*V* curves (Fig. S18b). The photovoltaic parameters of stretchable solar cells taking advantage of EGain–Ag particles as the bottom contact under the application of different strains are summarized in Table S1. After repeated cycles (stretching → releasing) of 1, 5, and 10 times, the performances of the stretchable solar cells composed of PEDOT:PSS/SiMW–PDMS composite/EGain–Ag particles were maintained without significant degradation (Fig. S19a and Table S2). Moreover, when each parameter was normalized against its initial value, there was no significant difference regarding *V*_oc_, *J*_sc_, FF and efficiency, indicating that stretchable solar cells are mechanically durable (Fig. S19b).

In summary, freestanding SiMPFs with adjustable transparency were fabricated for application in true-color transparent solar cells with flexibility. Novel wet etching was utilized to transform the morphology of the flat-top surface of the SiMPF to a slanted shape. Finite-difference time-domain simulation was used to elucidate the phenomena underlying the enhanced absorption, indicating re-absorption of light by adjacent SiMWs and an enhanced electric field in SiMWs with a slanted morphology. Furthermore, neutral-color transparent solar cells based on slanted-tip SiMPFs demonstrated a PCE of 8.07% at 10% average transmittance. Moreover, the platform is based on the c-Si wafer, which is already verified and widely used in the Si PV market. In addition, the freestanding platform is fabricated by photolithography and etching processes that are now widely used in the semiconductor industry; thus, it could be combined for advanced optics^87^, including microcavities and waveguides fabricated by lithography and etching for enhancing the performance. Finally, the transparent solar cell in this study is an ideal that can be utilized in real-world applications such as building-integrated photovoltaics, automobile attachable devices, or the Internet of things in the future.

## Materials and methods

### Fabrication of Si microwire arrays

Silicon microwire arrays were fabricated using *n*-Si wafers (Czochralski-grown, 525 ± 25-μm thick, 0.01–0.02 Ω cm, Unisill Inc.). A 20-nm-thick conformal SiO_2_ oxide layer was grown by dry thermal oxidation for 30 min at 1000 °C under a pure O_2_ atmosphere. Thereafter, a 70-nm SiO_2_ thin film was deposited by plasma-enhanced chemical vapor deposition (PEH-600, Sorona). Circular dot arrays (2 μm in diameter, 4, 5, 6, and 7 μm center-to-center distance) were patterned on the Si substrate by image reversal photolithography with an AZ5214 (AZ Electronic Materials) instrument. After deposition of Cr (300 nm) on the patterned Si photoresist, the residue was removed with acetone, resulting in Cr microdot arrays as a metal mask for the Si microwires. The Si was then etched by deep-reactive ion etching (DRIE) (Tegal 200). The DRIE process was carried out using SF_6_ (250 sccm)/C_4_F_8_ (150 sccm) in cyclic etching mode and passivation with a 1500 W source power, using 40 mTorr gas pressure and 100 W stage power. The Cr metal mask was removed with a Cr etchant after the DRIE process.

### Fabrication of Si microwire array/PDMS

A 15-nm-thick Al_2_O_3_ layer was deposited on the Si microwire arrays by atomic layer deposition (Lucida D100, NCD) to passivate the Si surface. The Al_2_O_3_-coated Si microwire arrays were treated with O_2_ plasma by reactive ion etching (RIE, Labstar) for a uniform coating with PDMS. The microwire arrays were then coated with a solution containing 5 g hexamethylcyclotrisiloxane (Sigma Aldrich), 1 g PDMS (Sylgard 184, Dow Corning), 0.10 g PDMS curing agent, and 5 ml dichloromethane. The solution was spin-coated onto the samples at 800 rpm for 120 s and 1200 rpm for 10 s, and cured at 100 °C for 20 min. PDMS etching was performed to expose the tip of the Si microwires via RIE at an RF power of 500 W. The samples were placed into the chamber at a pressure of 60 mTorr; the total gas flow rate was 80 sccm, with O_2_ gas and SF_6_ gas flow rates of 50 and 30 sccm, respectively.

### Fabrication of freestanding slanted-tip SiMPF

After dry etching of residual PDMS, only the upper part of the Al203-coated Si microwires is exposed. Apart from this, the filled PDMS remains in the Al_2_O_3_-coated Si microwires and prevents the etching of Al_2_O_3_ in the other part. After that, the exposed Al_2_O_3_ layers on the upper part of the Si microwires were etched with 85% H_3_PO_4_ at 50 °C for 180 s. Thereafter, the Si microwire arrays were chemically etched with a solution comprising HF/HNO_3_/CH_3_COOH/DMF in a volume ratio of 3:6:0.3:0.7. The detailed and overall etching process to fabricate the slanted-tip Si microwire–PDMS composite film is described in Fig. S20. The Si microwire array–PDMS composite film was mechanically peeled off from the parent substrate. For proper contact with the bottom electrodes and top junction layer, the composites were slightly etched with a 1:2 mixture of 1.0 M tetrabutylammonium fluoride in tetrahydrofuran (Sigma Aldrich) and dimethylformamide for 10 s. A 200-nm-thick indium-doped ZnO thin film was coated onto the composite films as a bottom electrode by RF sputtering (Infovion). A highly conductive PEDOT:PSS (CLEVIOS PH 1000) solution containing 9 wt% ethylene glycol and 0.1 wt% Triton X-100 was spin-coated onto the composite films to form the junction.

### Optical simulation of Si microwire arrays

Numerical simulation was performed with lumerical finite-difference time-domain (FDTD) as the time-dependent Maxwell's equation solver. We used material data from the literature to provide the refractive index information for Si, PDMS, and Al_2_O_3_. As a condition for the simulation of the incident light, a plane-wave parallel to the microwire was used. As the boundary condition for the simulation, Bloch boundary conditions were used in the *x* and *y* directions, and the perfect matched layer boundary was used in the *z* direction. For the absorption and reflection spectra and time-resolved reflected light of the microwire, the simulations were performed using the same parameters as those employed in the experiments.

### Characterization of transparent solar cells

The morphologies of the Si microwires and composites were characterized by field-emission scanning electron microscopy (Quanta200 FE-SEM, FEI). The transmittance and reflectance of the Si microwire–PDMS composite films were measured by using a UV–Vis–NIR spectrophotometer (Cary 5000, Agilent) with an integrating sphere to account for the total diffuse and specular light reflected and transmitted from the samples. The color coordinates of the samples were recorded with a goniometer (Neolight G500, PIMAX) equipped with a compact array spectrometer (CAS 140 CT, Instrument system) using 1-sun illumination. The external quantum efficiency spectra were recorded in the wavelength range 400–1100 nm using a xenon light source and a monochromator. The active area of the devices equalled 0.5 × 0.5 cm^2^. A shadow mask with an area of 0.25 cm^2^ was also used for precisely defining the active area. The solar cell results were therefore referenced to the active area. The photovoltaic performance of the solar cells was examined under AM 1.5 G illumination using a solar simulator. The incident flux was confirmed using a NREL-calibrated solar cell (PV Measurements, Inc.)

## Supplementary information

SUPPLEMENTARY INFORMATION for Stretchable and colorless freestanding microwire arrays for transparent solar cells with flexibility

Supplementary Movie S1
