# Supplementary material for: Stretchable and colorless freestanding microwire arrays for transparent solar cells with flexibility
Source: Light Sci Appl. 2019 Dec 12;8:121. doi: 10.1038/s41377-019-0234-y (PMC6908716; doi:10.1038/s41377-019-0234-y)
Supplement: Supplementary file 1 — SUPPLEMENTARY INFORMATION for Stretchable and colorless freestanding microwire arrays for transparent solar cells with flexibility [file 41377_2019_234_MOESM1_ESM.docx]

**Stretchable and colorless freestanding microwire arrays for transparent solar cells with flexibility**

Sung Bum Kang^1^, Ji-Hwan Kim^2^, Myeong Hoon Jeong^1^, Amit Sanger^1^, Chan Ul Kim^1^, Chil-Min Kim^2^ and Kyoung Jin Choi^1*^

^1^ Department of Materials Science and Engineering, Ulsan National Institute of Science and Technology (UNIST), Ulsan 44919, Republic of Korea. Correspondence and requests for materials should be addressed to K.J. C (email: [choi@unist.ac.kr](mailto:choi@unist.ac.kr))

^2^ Department of Emerging Materials Science, Daegu Gyeongbuk Institute of Science and Technology (DGIST), Daegu 42988, Republic of Korea.

**Fig. S1**. J-V characteristics of the transparent solar cells without Al_2_O_3_ passivation.

**Fig. S2**. (a,b) Cross sectional SEM images of Si microwire arrays filled with PDMS before the surface treatment

**Fig. S3.** Optical diffraction pattern produced by transmitted light (λ = 532 nm), demonstrating the long-range order of the wires.

**Fig. S4**. SEM images of Si microwire arrays; pitch of (a) 4 μm pitch, (b) 5 μm, (c) 6 μm and (d) 7 μm.

**Fig. S5**. Photovoltaic performances of TSCs based on flat SiMPFs (a) J-V characteristics and (b) EQE of the transparent solar cells with controlled pitches.

**Fig. S6.** Box plots (minimum–maximum, 25–75%, mean: open square symbol, median: solid line) of photovoltaic parameters: (a) short circuit current, (b) open circuit voltage and (c) efficiency of flat-tip of SiMPF-based solar cells.

**Fig. S7.** (a) The experimental and calculated reflectance spectra of planar Si (black square and 24 % of planar Si (black circle, calculated). (b) J-V characteristic of the TSC based on the 4 μm-pitched SiMW array with (black line) and without SiNx (red line).

**Fig. S8.** SEM images showing (a) the dislodged Si microwire from the PDMS due to aggressive HF:HNO_3_ (3:7) etching, (b) Etched Si microwire by the 3:6:1 (HF:HNO_3_:CH_3_COOH) solution and (c) by 3:6:1 (HF:HNO_3_:DMF) solution. (d) Slanted Si microwire by the solution of having a HF: HNO_3_: CH_3_COOH: DMF volume ratio of 3:6:0.3:0.7.

**Fig. S9.** Time resolved light-tracing simulation with wavelength of 550 nm at (a) flat and (b) slanted Si microwire.

**Fig. S10.** Time resolved light-tracing simulation with wavelength of 900 nm at (a) flat and (b) slanted Si microwire.

**Fig. S11.** The configurations to measure and calculate the haze ratio.

**Fig. S12.** (a) The haze of the SiMPF with flat (circle dot) and slanted (square dot) tip as a function of wavelength: 4 μm (green line), 5 μm (blue line), 6 μm (yellow line) and 7 μm (red line). (a) Schematic illustration of refracted and reflected light at the slanted microwire

**Fig. S13. (a)** Optical images of perovskite filters: i) MAPb(I_0.41_Br_0.59_)_3_ , ii) MAPb(I_0.28_Br_0.72_)_3_ and iii) MAPb(I_0.05_Br_0.95_)_3_ (b) absorbance and (c) transmittance spectra of various perovskite filters

**Fig. S14.** Box plots (minimum–maximum, 25–75%, mean: open square symbol, median: solid line) of photovoltaic parameters: (a) short circuit current, (b) open circuit voltage and (c) efficiency of slanted-tip of SiMPF-based solar cells.

**Fig. S15.** The EQE enhancement of slanted SiMPF based devices.

**Fig. S16.** The transmittance spectra of the sample with 5 um pitch without (red line) and with strain (black line).

**Fig. S17.** Sheet resistance versus strain of IZO (red line) and EGain-Ag particles electrodes (black line) on PDMS subjected to uniaxial strain.

**Fig. S18.** (a) Light J-V curves and (b) corresponded external quantum efficiency of stretchable solar cells under the different strain.

**Fig. S19.** (a) Light J-V curves and (b) Normalized photovoltaic parameters of stretchable solar cells before and after 5 and 10 cycles.

**Fig. S20.** Overall etching process to fabricate the slanted-tip of Si microwire – PDMS composite film.

**Table S1.** The photovoltaic parameters of stretchable solar cells taking advantages of EGain –Ag particles as bottom contact under the application of different strain.

**Table S2.** The photovoltaic parameters of stretchable solar cells taking advantages of EGain –Ag particles as bottom contact after repeated stretching – releasing cycles.


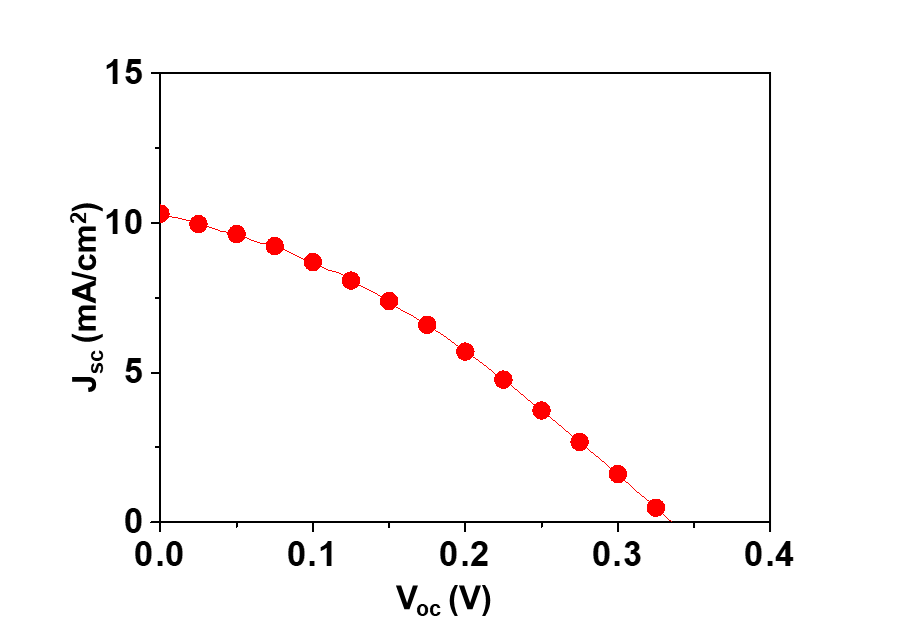


|  | ***V*_oc_**  **(V)** | ***J*_sc_**  **(mA/cm^2^)** | **FF** | **Eff.**  **(%)** |
| --- | --- | --- | --- | --- |
| **W/O Al_2_O_3_ passivation** | 0.33 | 10.7 | 0.327 | **1.08** |

**Fig. S1**. J-V characteristics of the transparent solar cells without Al_2_O_3_ passivation.


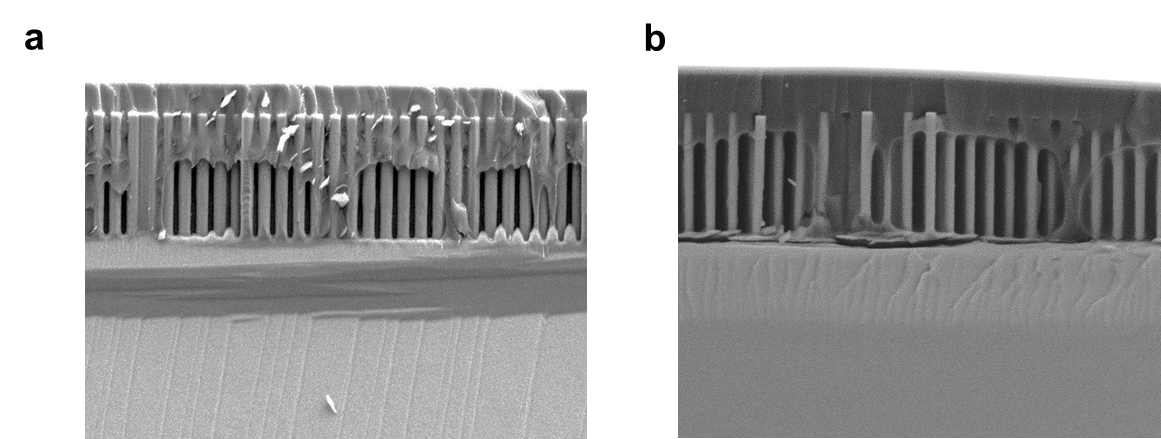


**Fig. S2**. (a,b) Cross sectional SEM images of Si microwire arrays filled with PDMS before the surface treatment


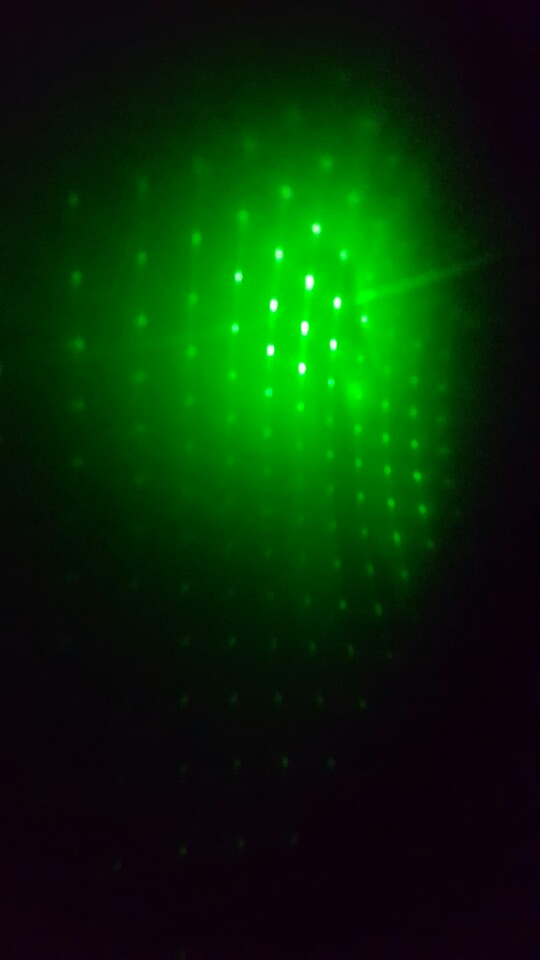


**Fig. S3.** Optical diffraction pattern produced by transmitted light (λ = 532 nm), demonstrating the long-range order of the wires.


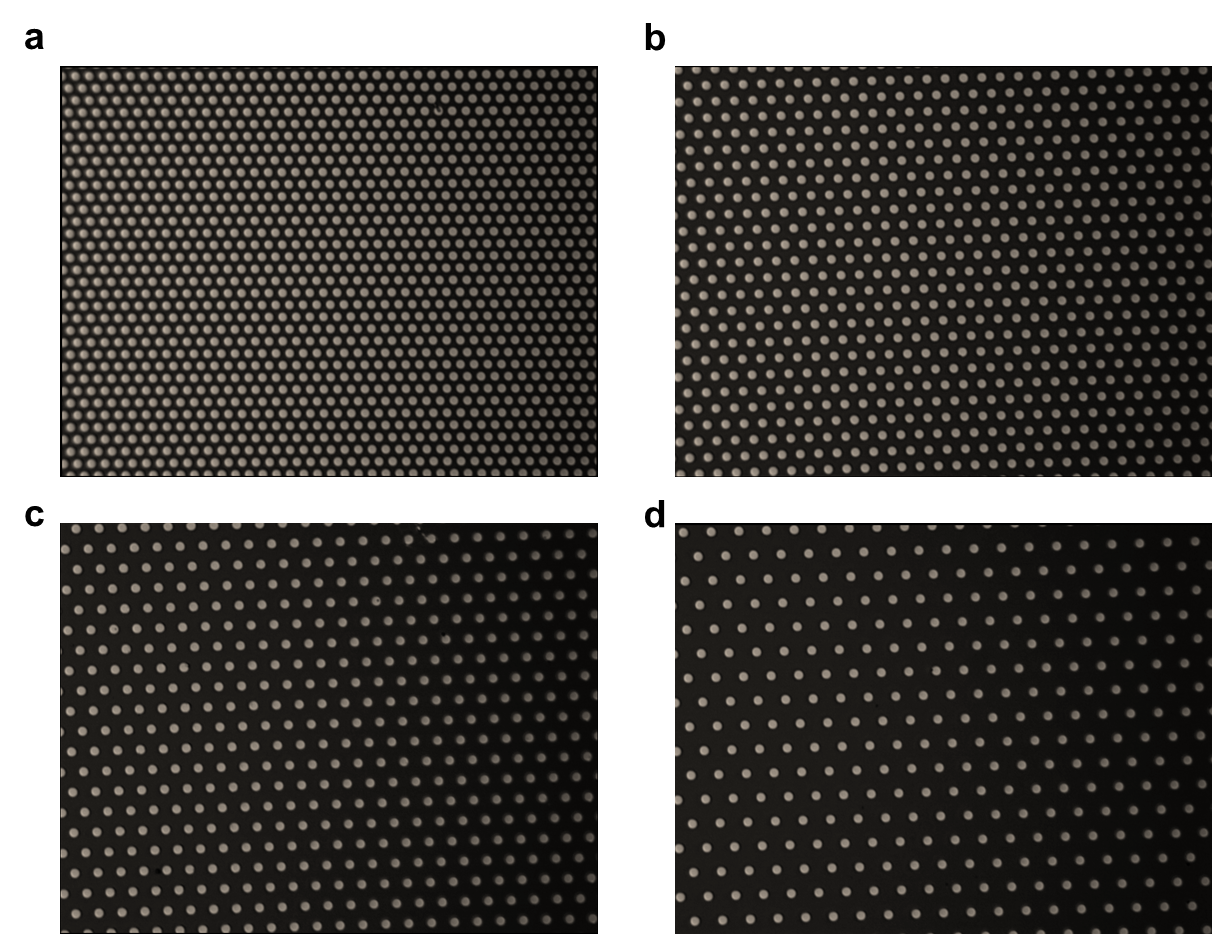


**Fig. S4**. SEM images of Si microwire arrays; pitch of (a) 4 μm pitch, (b) 5 μm, (c) 6 μm and (d) 7 μm.


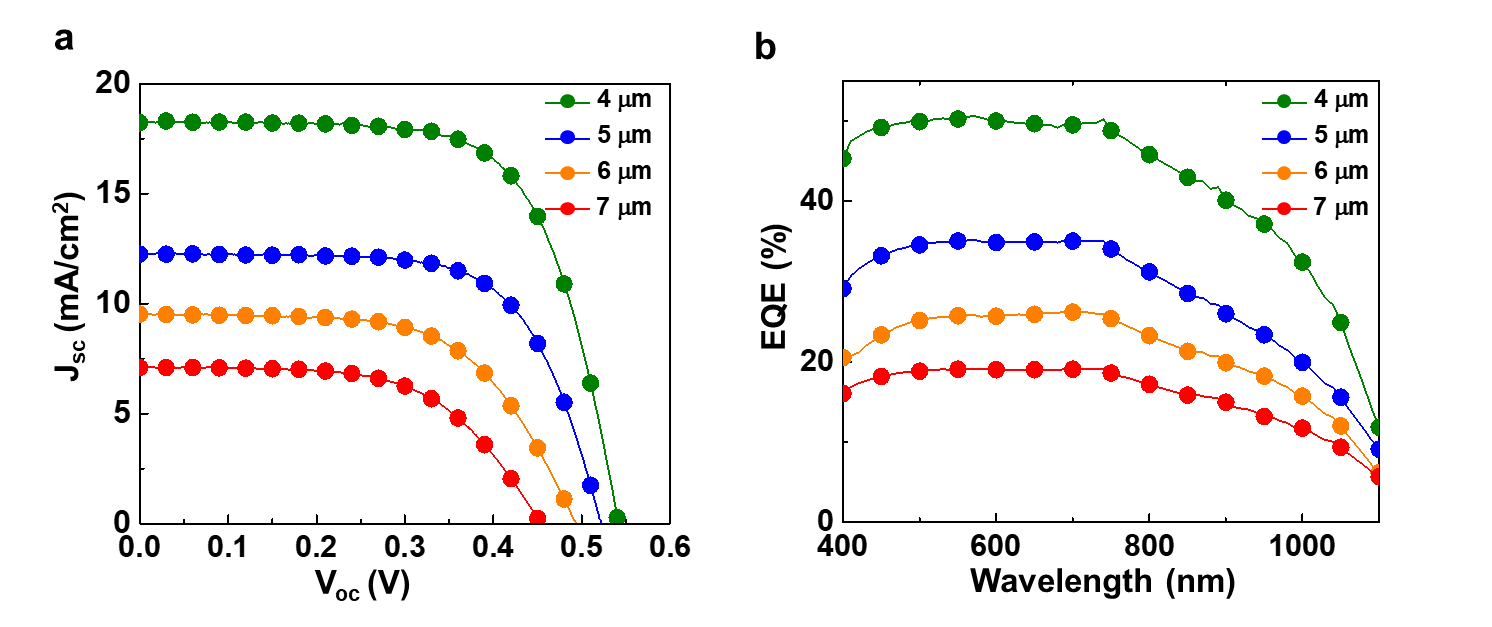


**Fig. S5**. Photovoltaic performances of TSCs based on flat SiMPFs (a) J-V characteristics and (b) EQE of the transparent solar cells with controlled pitches.


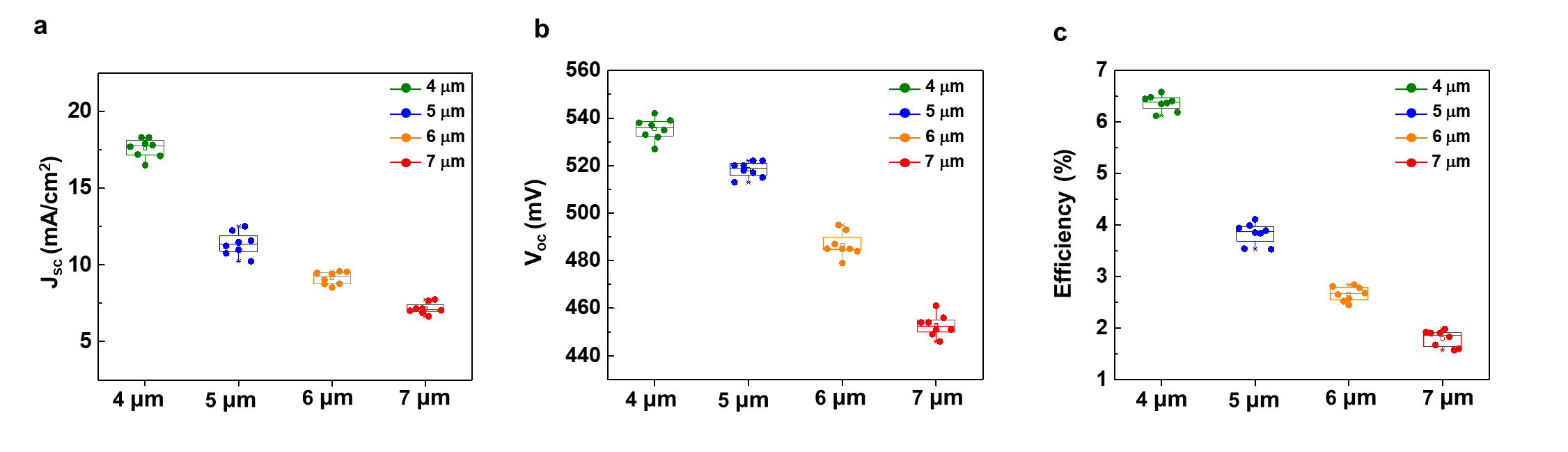
**Fig. S6.** Box plots (minimum–maximum, 25–75%, mean: open square symbol, median: solid line) of photovoltaic parameters: (a) short circuit current, (b) open circuit voltage and (c) efficiency of flat-tip of SiMPF-based solar cells.


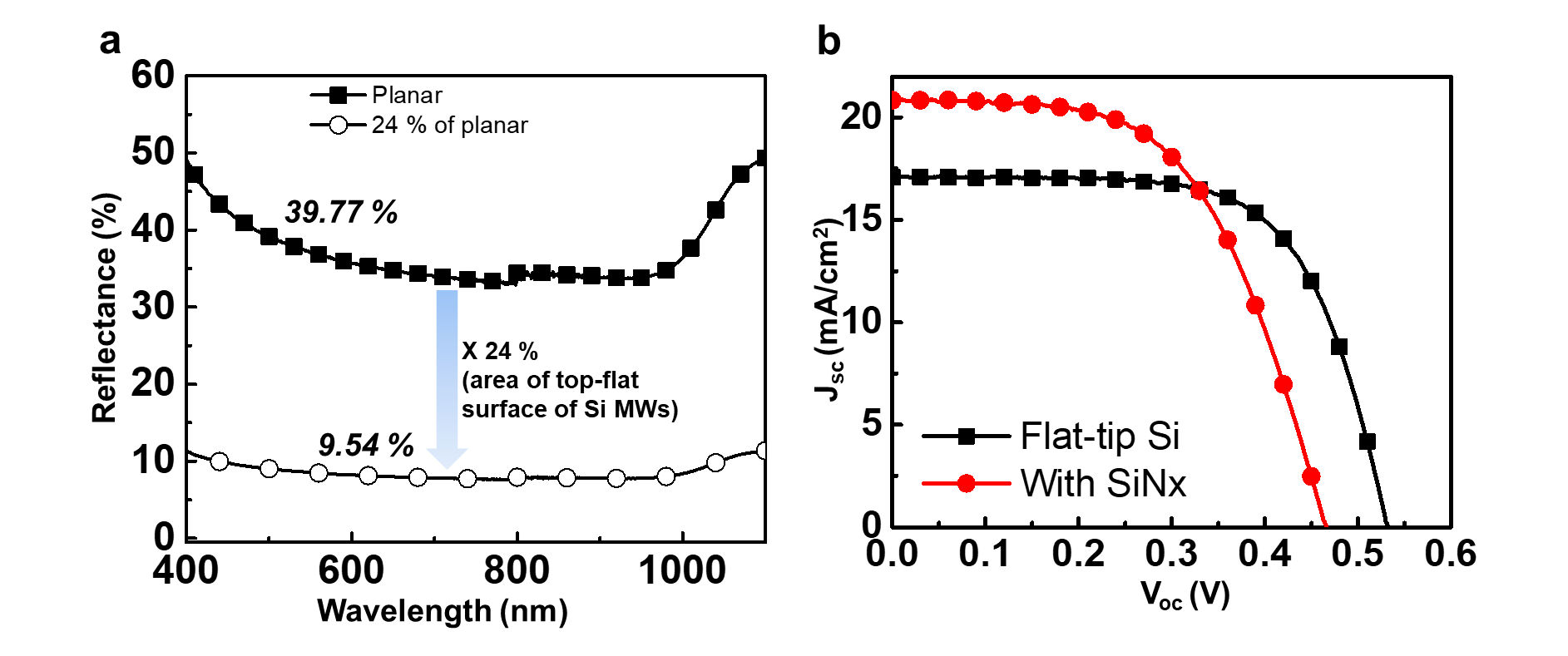


|  | ***V*_oc_**  **(V)** | ***J*_sc_**  **(mA/cm^2^)** | **FF** | **Eff.**  **(%)** |
| --- | --- | --- | --- | --- |
| **Flat tip Si** | 0.533 | 17.07 | 0.658 | **5.99** |
| **With SiNx on the tip** | 0.466 | 18.94 | 0.534 | **4.71** |

**Fig. S7.** (a) The experimental and calculated reflectance spectra of planar Si (black square and 24 % of planar Si (black circle, calculated). (b) J-V characteristic of the TSC based on the 4 μm-pitched SiMW array with (black line) and without SiNx (red line).


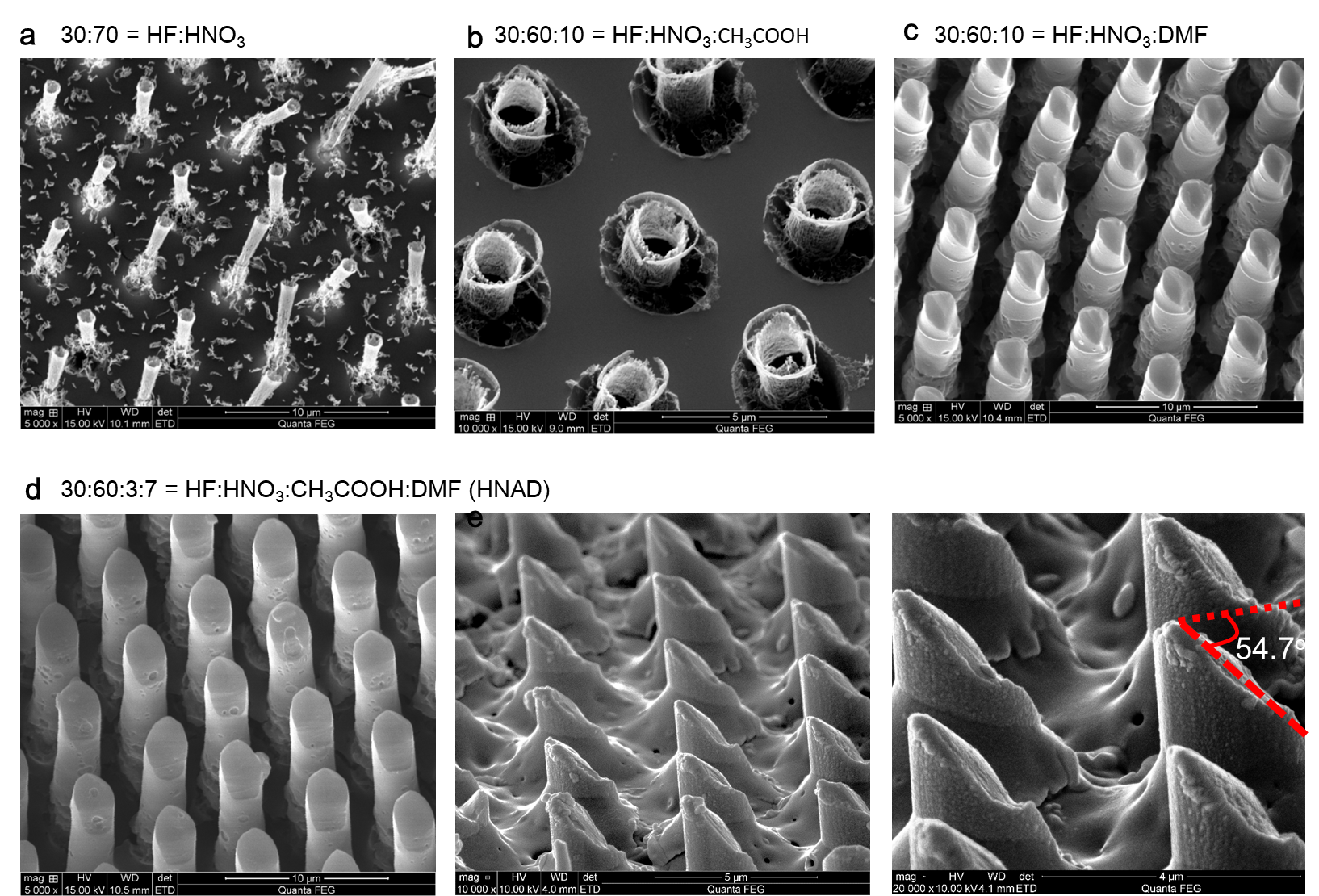


**Fig. S8.** SEM images showing (a) the dislodged Si microwire from the PDMS due to aggressive HF:HNO_3_ (3:7) etching, (b) Etched Si microwire by the 3:6:1 (HF:HNO_3_:CH_3_COOH) solution and (c) by 3:6:1 (HF:HNO_3_:DMF) solution. (d) Slanted Si microwire by the solution of having a HF: HNO_3_: CH_3_COOH: DMF volume ratio of 3:6:0.3:0.7.


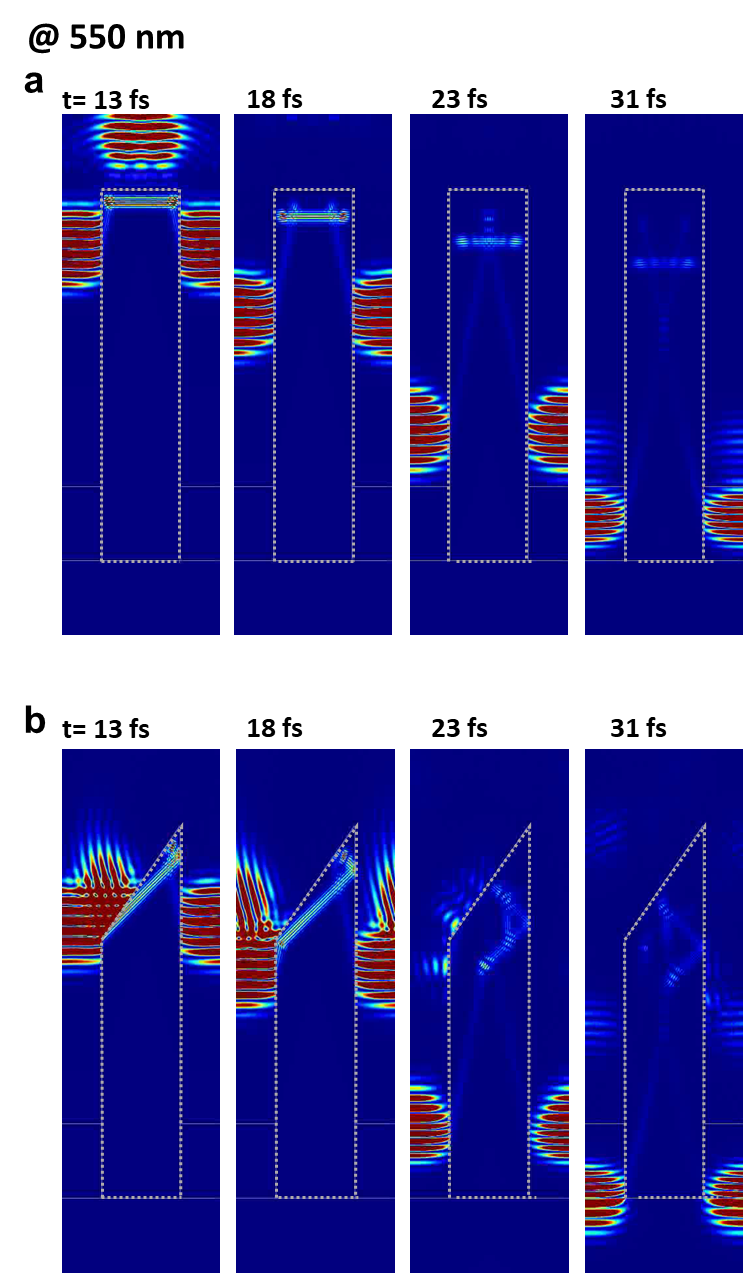


**Fig. S9.** Time resolved light-tracing simulation with wavelength of 550 nm at (a) flat and (b) slanted Si microwire.


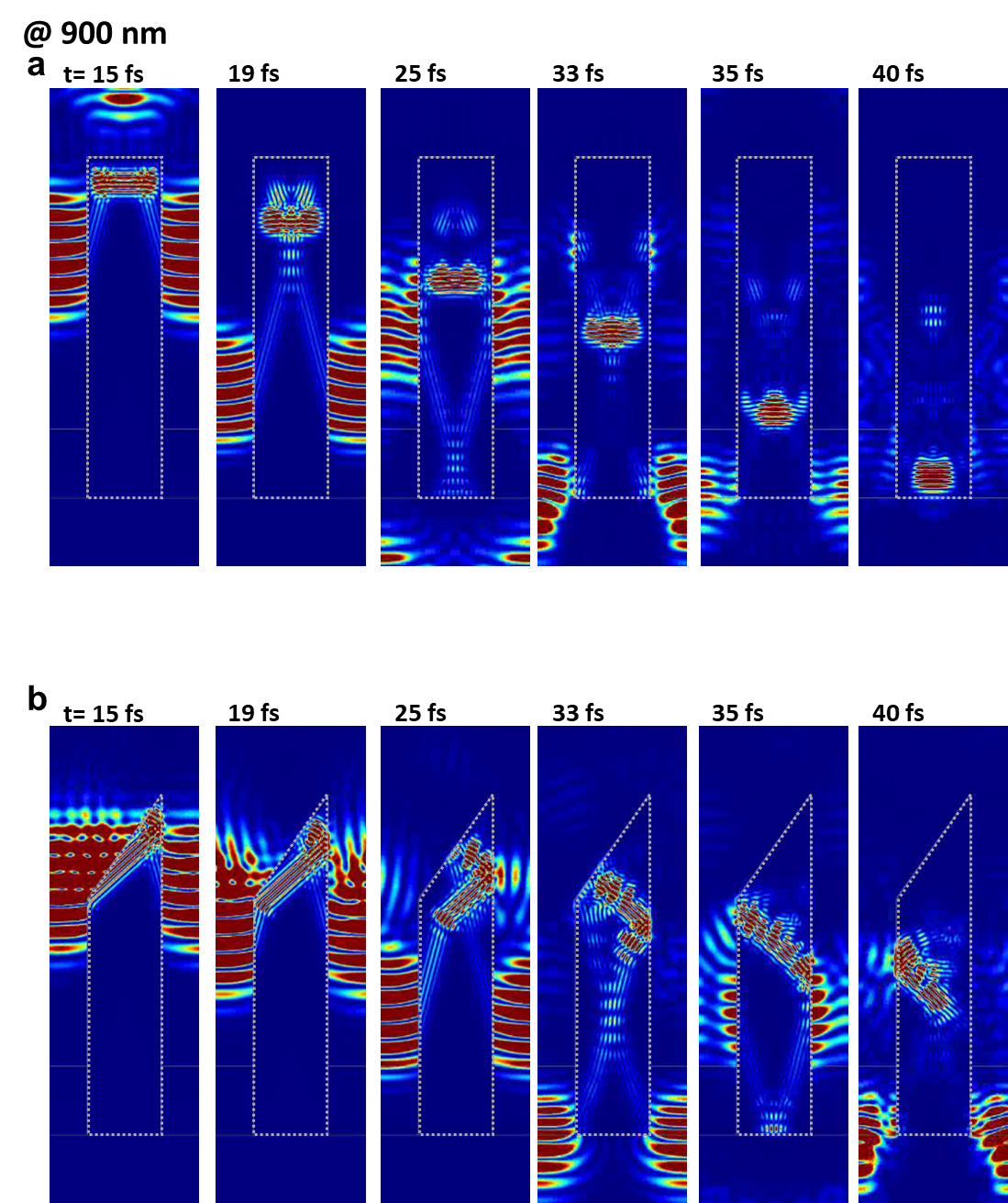


**Fig. S10.** Time resolved light-tracing simulation with wavelength of 900 nm at (a) flat and (b) slanted Si microwire.


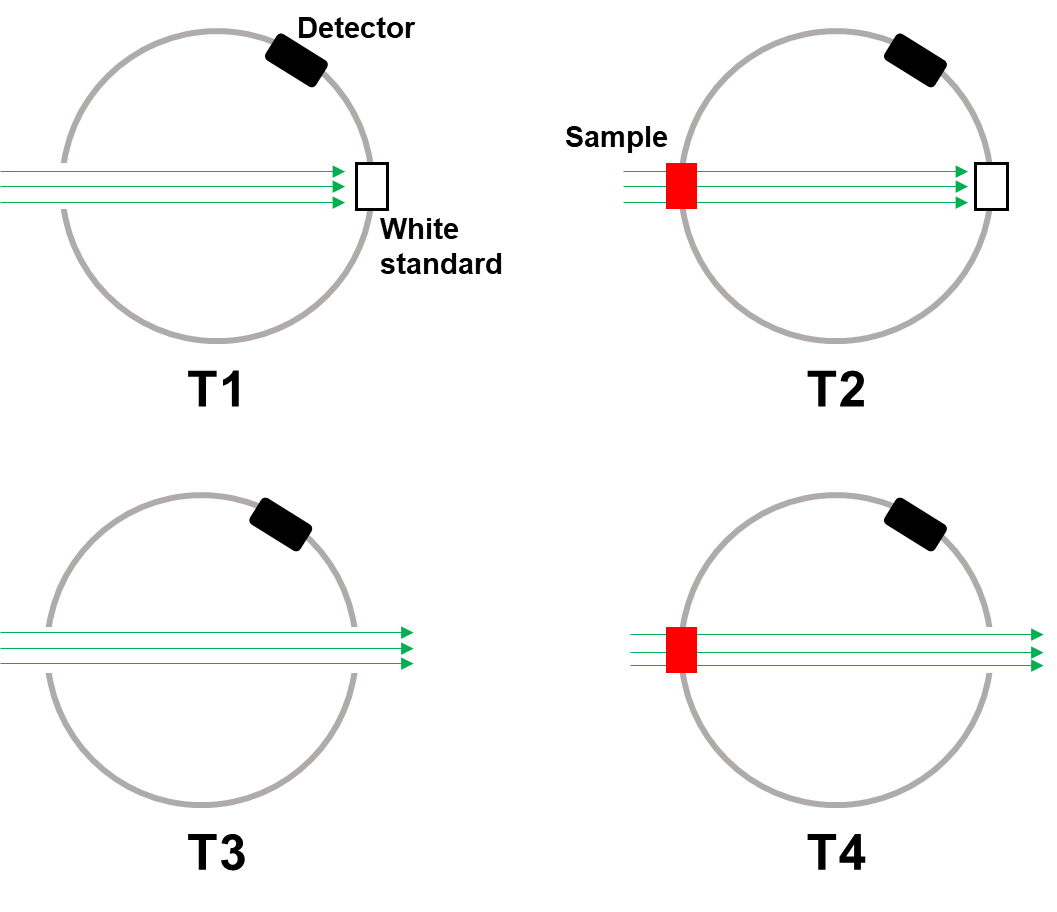


**Fig. S11.** The configurations to measure the haze ratio.

For each samples, the hazeness is calculated by four scans using the configuration as shown in Fig. Sxx. The haze value is defined as the ratio of *T*_diffuse_ (diffuse transmittance) *T*_total_ (total transmittance). At the configuration T1, transmittance is measured when integrating sphere is closed with white standard, while in configuration T2 additionally a sample is placed at the entrance to the sphere. In configuration T3 light can pass through the sphere undisturbed and in configuration T4 it is scattered by the sample at the entrance to the sphere The T1 and T3 are the corrections for the experimental setup. Moreover, T2 is total forward scattered illumination and T4 is total transmitted illumination, respectively. Therefore, the The haze (%) is T_diffuse_ / T_total_ where T_diffuse_ = [(T4 – T3*(T2/T1))]/T1 and T_total_ = T2/T1.


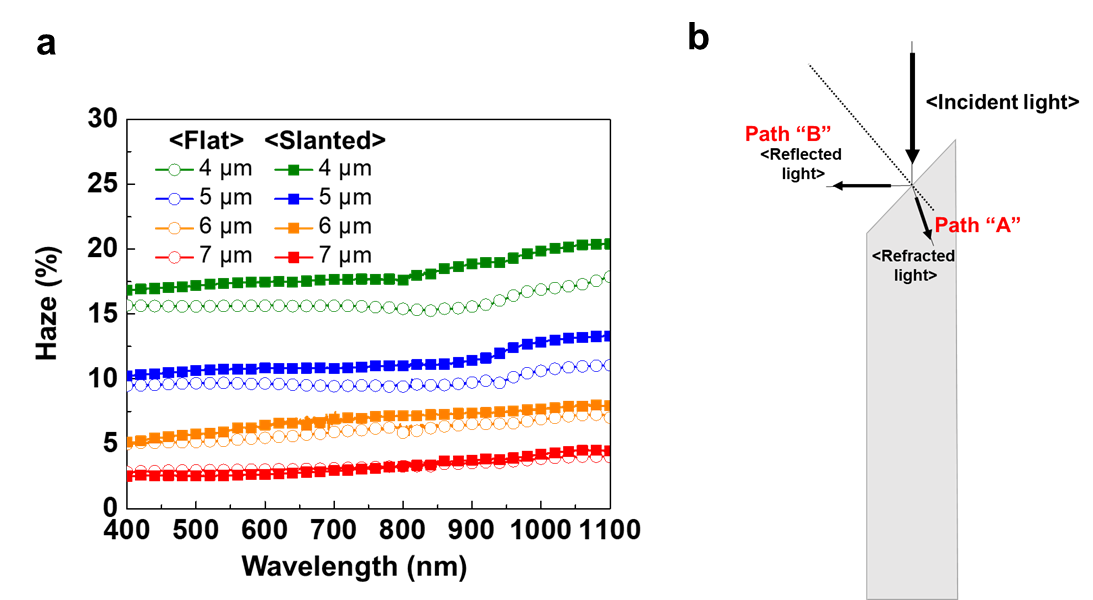
**Fig. S12.** (a) The haze of the SiMPF with flat (circle dot) and slanted (square dot) tip as a function of wavelength: 4 μm (green line), 5 μm (blue line), 6 μm (yellow line) and 7 μm (red line). (a) Schematic illustration of refracted and reflected light at the slanted microwire


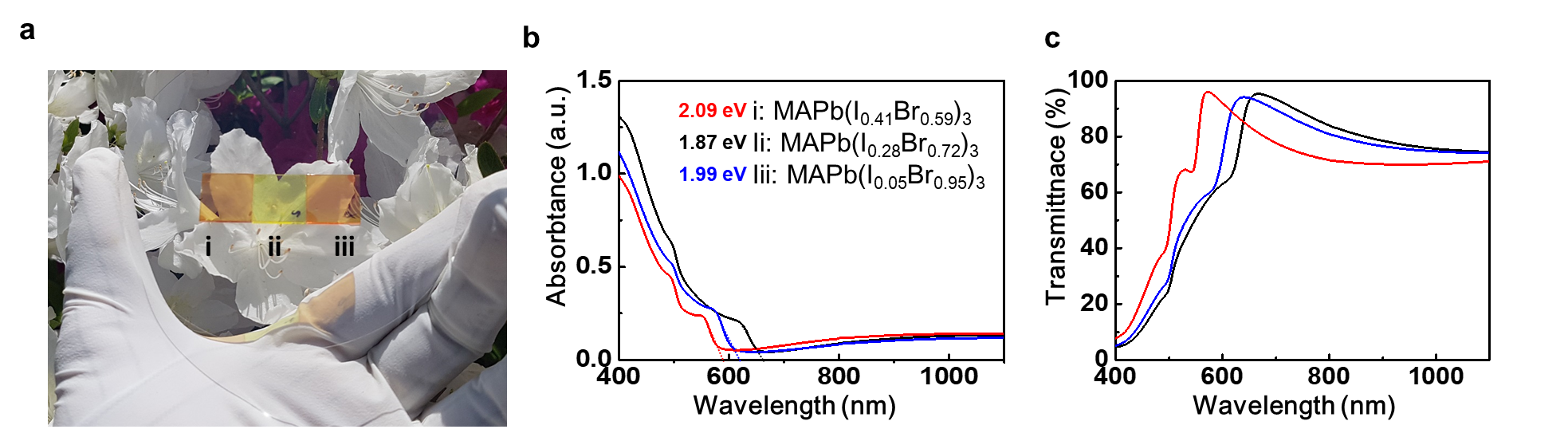


**Fig. S13. (a)** Optical images of perovskite filters: i) MAPb(I_0.41_Br_0.59_)_3_ , ii) MAPb(I_0.28_Br_0.72_)_3_ and iii) MAPb(I_0.05_Br_0.95_)_3_ (b) absorbance and (c) transmittance spectra of various perovskite filters


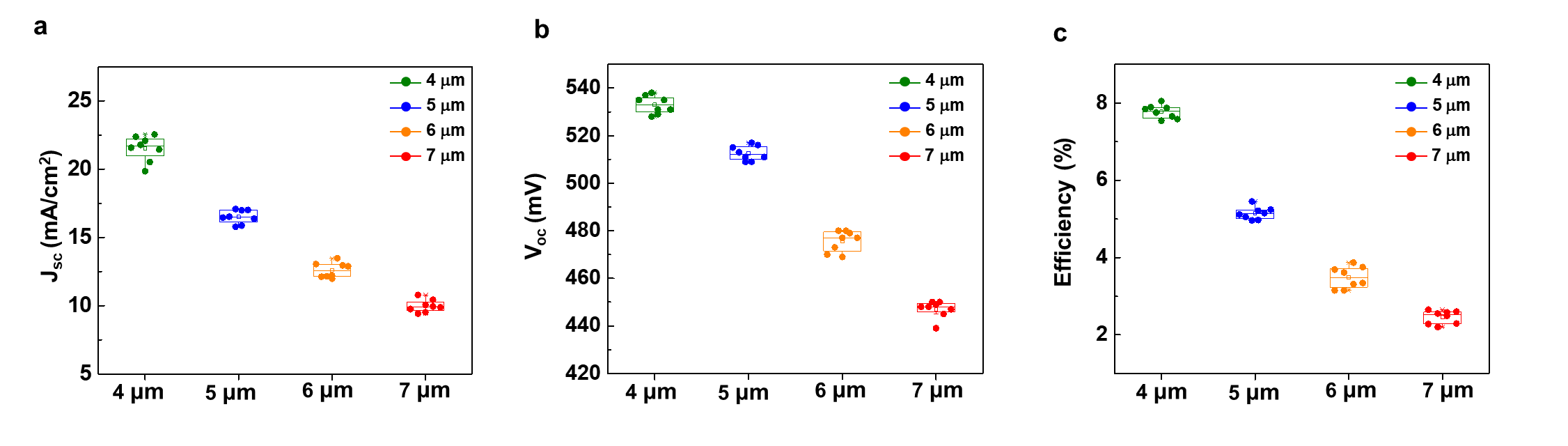
**Fig. S14.** Box plots (minimum–maximum, 25–75%, mean: open square symbol, median: solid line) of photovoltaic parameters: (a) short circuit current, (b) open circuit voltage and (c) efficiency of slanted-tip of SiMPF-based solar cells.


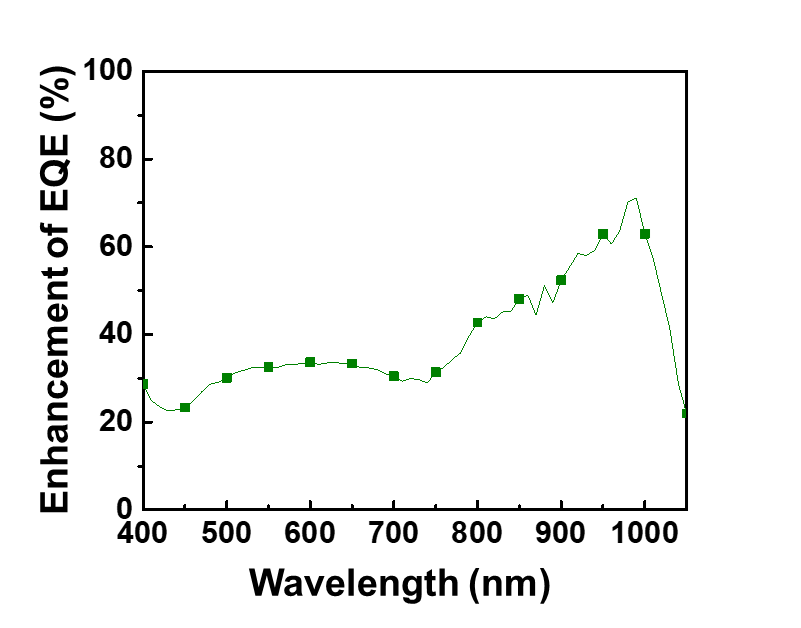


**Fig. S15.** The averaged EQE enhancement of slanted SiMPF based devices as function of wavelength.


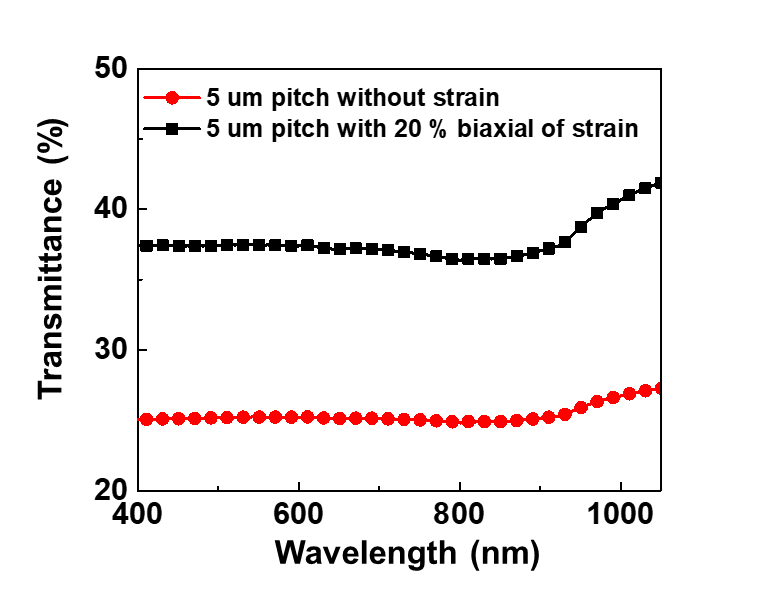


**Fig. S16.** The transmittance spectra of the sample with 5 um pitch without (red line) and with strain (black line).


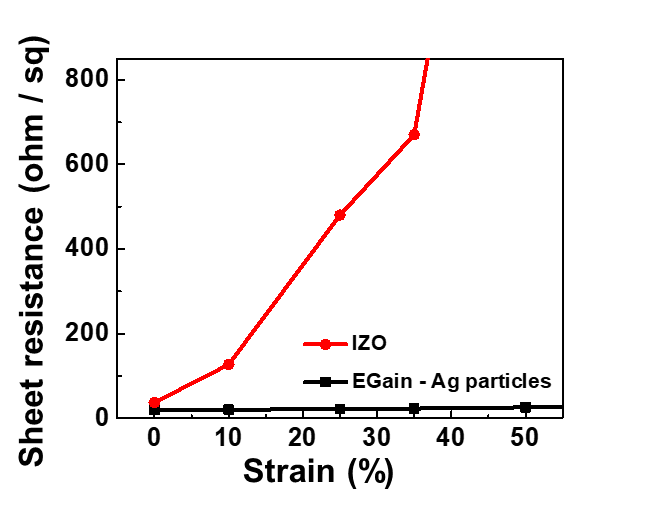


**Fig. S17.** Sheet resistance versus strain of IZO (red line) and EGain-Ag particles electrodes (black line) on PDMS subjected to uniaxial strain.


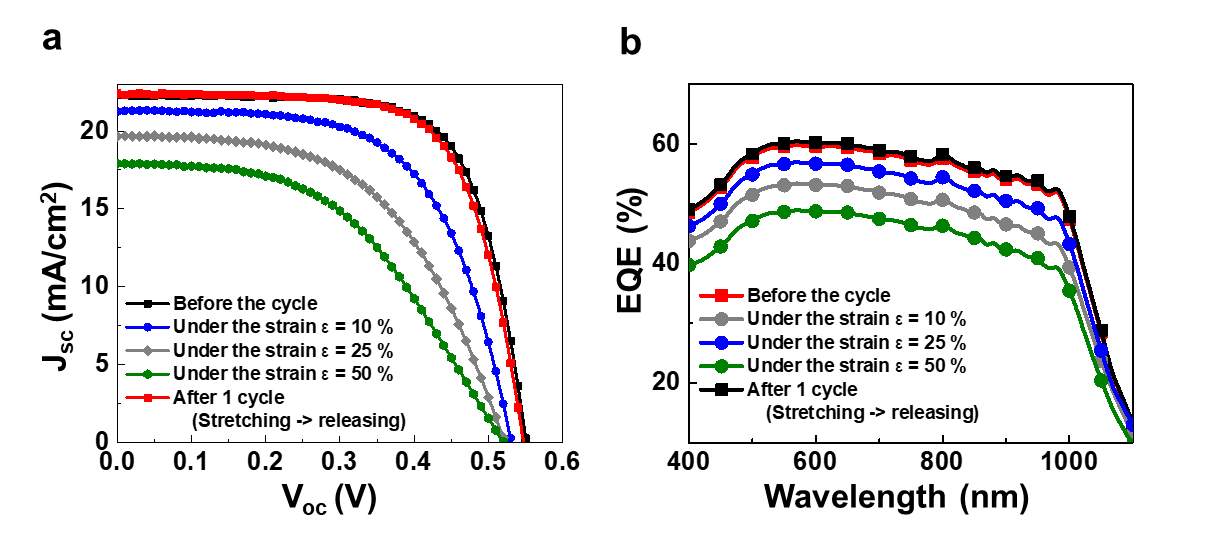
**Fig. S18.** (a) Light J-V curves and (b) corresponded external quantum efficiency of stretchable solar cells under the different strain.

**Table S1.** The photovoltaic parameters of stretchable solar cells taking advantages of EGain –Ag particles as bottom contact under the application of different strain.

| **Applied strain** | ***V*_oc_**  **(V)** | ***J*_sc_**  **(mA/cm^2^)** | **FF** | **Eff.**  **(%)** |
| --- | --- | --- | --- | --- |
| **ε = 0 %**  **(Before the cycle)** | 0.537 | 22.289 | 0.698 | **8.360** |
| **Under the ε = 10 %** | 0.531 | 21.258 | 0.614 | **6.933** |
| **Under the ε = 25 %** | 0.522 | 19.641 | 0.538 | **5.520** |
| **Under the ε = 50 %** | 0.520 | 17.916 | 0.483 | **4.497** |
| **ε = 0 %**  **After 1 cycle (Stretching -> Releasing)** | 0.533 | 22.381 | 0.686 | **8.179** |


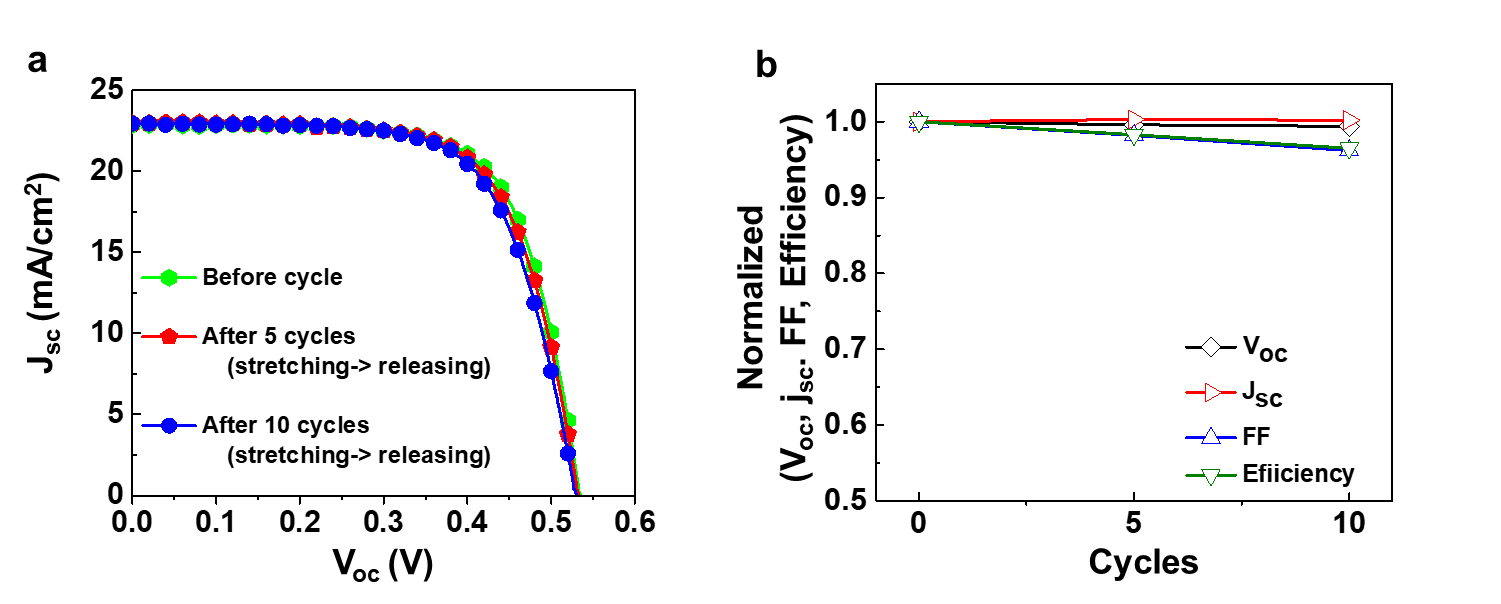
**Fig. S19.** (a) Light J-V curves and (b) Normalized photovoltaic parameters of stretchable solar cells before and after 5 and 10 cycles.

|  | ***V*_oc_**  **(V)** | ***J*_sc_**  **(mA/cm^2^)** | **FF** | **Eff.**  **(%)** |
| --- | --- | --- | --- | --- |
| **Before cycle** | 0.534 | 22.86 | 0.697 | **8.51** |
| **After 5 cycles** | 0.532 | 22.94 | 0.685 | **8.37** |
| **After 10 cycles** | 0.531 | 22.92 | 0.673 | **8.19** |

**Table S2.** The photovoltaic parameters of stretchable solar cells taking advantages of EGain –Ag particles as bottom contact after repeated stretching – releasing cycles.

**
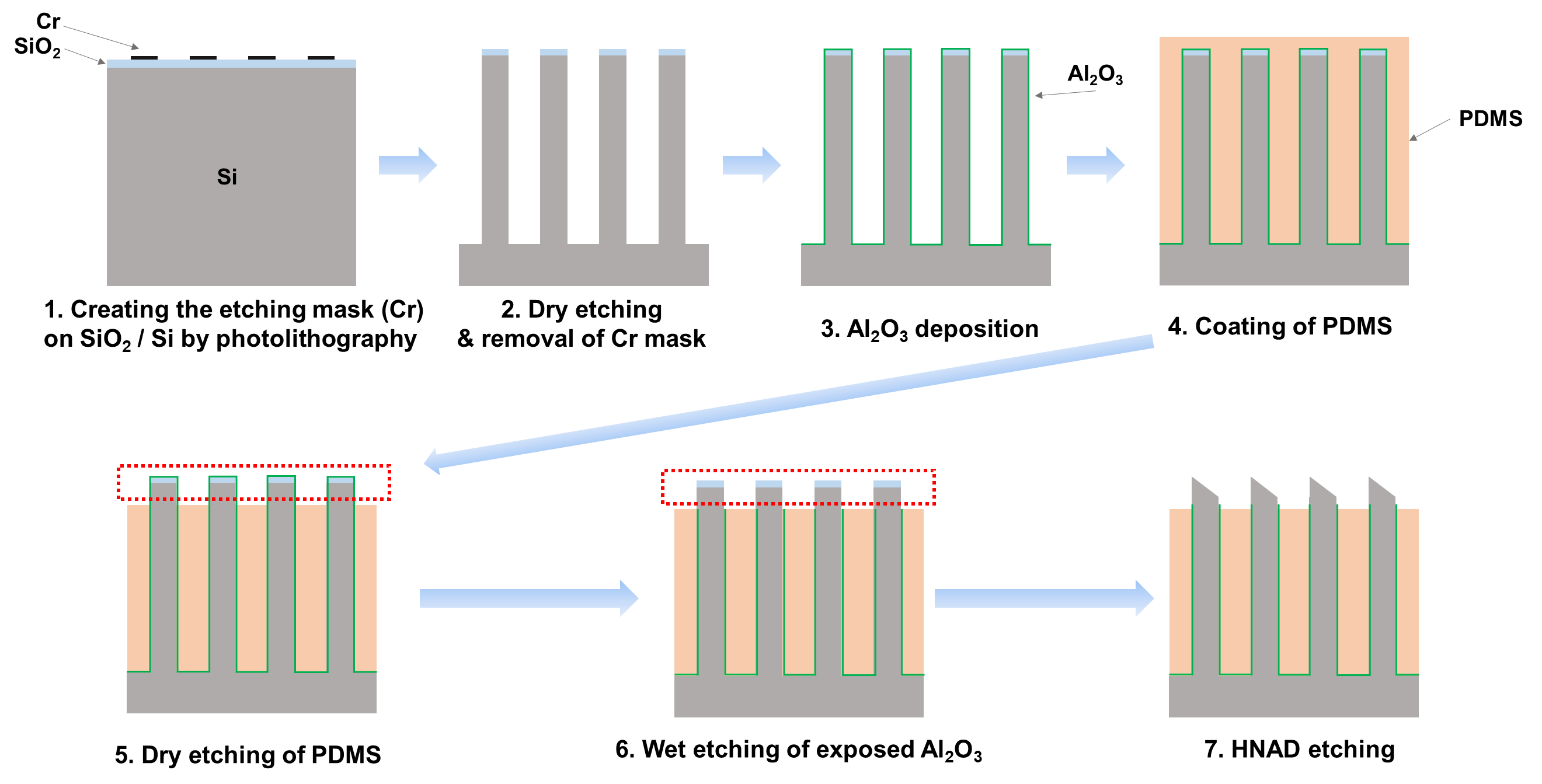
Fig. S20.** Overall etching process to fabricate the slanted-tip of Si microwire – PDMS composite film.
